# Assistive Loading Promotes Goal-Directed Tuning of Stretch Reflex Gains

**DOI:** 10.1523/ENEURO.0438-22.2023

**Published:** 2023-02-23

**Authors:** Frida Torell, Sae Franklin, David W. Franklin, Michael Dimitriou

**Affiliations:** 1 Physiology Section, Department of Integrative Medical Biology, Umeå University, S-901 87 Umeå, Sweden; 2 Neuromuscular Diagnostics, Department of Sport and Health Sciences, Technical University of Munich, D-80992 Munich, Germany; 3 Munich Institute of Robotics and Machine Intelligence (MIRMI), Technical University of Munich, D-80992 Munich, Germany; 4 Munich Data Science Institute (MDSI), Technical University of Munich, 85748 Munich, Germany

**Keywords:** assistive loading, goal-directed, movement preparation, reaching, stretch reflex

## Abstract

Voluntary movements are prepared before they are executed. Preparatory activity has been observed across the CNS and recently documented in first-order neurons of the human PNS (i.e., in muscle spindles). Changes seen in sensory organs suggest that independent modulation of stretch reflex gains may represent an important component of movement preparation. The aim of the current study was to further investigate the preparatory modulation of short-latency stretch reflex responses (SLRs) and long-latency stretch reflex responses (LLRs) of the dominant upper limb of human subjects. Specifically, we investigated how different target parameters (target distance and direction) affect the preparatory tuning of stretch reflex gains in the context of goal-directed reaching, and whether any such tuning depends on preparation duration and the direction of background loads. We found that target distance produced only small variations in reflex gains. In contrast, both SLR and LLR gains were strongly modulated as a function of target direction, in a manner that facilitated the upcoming voluntary movement. This goal-directed tuning of SLR and LLR gains was present or enhanced when the preparatory delay was sufficiently long (>250 ms) and the homonymous muscle was unloaded [i.e., when a background load was first applied in the direction of homonymous muscle action (assistive loading)]. The results extend further support for a relatively slow-evolving process in reach preparation that functions to modulate reflexive muscle stiffness, likely via the independent control of fusimotor neurons. Such control can augment voluntary goal-directed movement and is triggered or enhanced when the homonymous muscle is unloaded.

## Significance Statement

It is well known that movement preparation improves motor performance. That is, briefly delaying the onset of a goal-directed movement can significantly benefit the overall quality of movement. However, the mechanisms underlying movement preparation remain unclear. In this study, we examined the preparatory modulation of short-latency and long-latency stretch reflex responses in the dominant upper limb. We found that goal-directed tuning of stretch reflex gains is consistently triggered or enhanced in cases where preparation is sufficiently long (>250 ms) and a background (“assistive”) load is first applied in the direction of homonymous muscle action. A better understanding of movement preparation will likely also benefit the development of rehabilitation regimes and movement augmentation devices.

## Introduction

Voluntary movements undergo preparation before their execution (Kutas and Donchin, 1974; Wise, 1985; Ghez et al., 1997). That is, voluntary movement normally involves a period of preparatory tuning where neural activity is functionally adjusted before movement initiation. This preparatory activity in the CNS correlates well with parameters such as movement direction (Tanji and Evarts, 1976), reach distance (Messier and Kalaska, 2000), movement speed (Churchland et al., 2006), reach trajectory (Hocherman and Wise, 1991), and visual target location (Batista et al., 2007). Motor preparation has long been thought of as the assembly of motor subroutines for later execution (Sternberg et al., 1978) or underlying the computation of appropriate timing and force level in the muscles tasked with achieving the motor goal (Brooks, 1979). Reaching movements involve the formation of feedforward motor commands and as well as feedback policy that modulates long-latency stretch reflex responses (LLRs) before movement onset (Todorov and Jordan, 2002; Shadmehr and Krakauer, 2008; Wagner and Smith, 2008; Ahmadi-Pajouh et al., 2012; Yeo et al., 2016).

Stretching of muscles in the upper extremities produces a short-latency reflex response (SLR) beginning ∼20–25 ms after stretch onset, and an LLR beginning 50 ms after stimulus onset (Hammond, 1956; Marsden et al., 1972). Both the SLR and the LLR are considered involuntary, with voluntary control of upper extremities beginning ∼100 ms after onset of the triggering sensory event (Yang et al., 2011). It has long been known that stretch reflex responses can allow for effective resistance against unwanted postural perturbations (Nichols and Houk, 1976). However, reflex responses can also be modulated subconsciously to accommodate the execution of voluntary movements. For example, in tasks involving active reaching, significant modulation of reflex responses have been observed according to target shape (Nashed et al., 2012), obstacles (Nashed et al., 2014), static and moving targets (Cluff and Scott, 2015), and target cue direction (Pruszynski et al., 2008; for review, see Scott, 2016).

In the upper extremities, the SLR is often characterized by automatic gain scaling (i.e., a preload sensitivity), whereas the LLR is found to be robustly task dependent (Pruszynski et al., 2009; 2011a). More recently, the early LLR (R2) has been shown to contain a stabilizing component modulated independently of the voluntary action planned in relation to a queued target, while the late LLR (R3) displayed task dependency (Lee and Perreault, 2019). Such studies reinforce the idea that at least certain components of the LLR are highly adaptable and not strictly “reflex” in nature (Shemmell et al., 2010). As mentioned above, goal-directed feedback controllers affecting LLRs are thought to be already loaded during the reach preparation phase (Ahmadi-Pajouh et al., 2012). However, it is still unclear what specific mechanism produces this tuned reflex modulation before movement initiation.

It is currently believed that cortical preparatory activity serves a dynamic system that defines the initial conditions for the progression of goal-directed movement (Churchland et al., 2010, 2012). However, exactly how cortical preparation manifests as improved motor performance is unclear. Recent findings propose one more specific neural mechanism in reach preparation. That is, movement preparation appears to include the independent and goal-directed tuning of muscle spindles, leading to a congruent modulation of reflex gains (Papaioannou and Dimitriou, 2021). These findings suggest that two independent output mechanisms are involved in the preparation of voluntary reaching (i.e., one involving the direct control of α motor neurons, and another implicating independent γ motor control; Dimitriou, 2021, 2022). By modulating the sensitivity of muscle spindles and stretch reflexes during preparation, the nervous system can adjust sensory feedback and reflex muscle stiffness independently of any coinciding muscle force during preparation. Our previous work has shown that target direction affects preparatory tuning of muscle spindles and stretch reflex gains in a manner that facilitates the upcoming voluntary movement (Papaioannou and Dimitriou, 2021). In the current study, we hypothesized that both target direction and target distance may impact the preparatory tuning of stretch reflex gains. In particular, here we examine whether the fundamental parameter of target distance affects preparatory tuning of stretch reflex gains in the context of goal-directed reaching, and how any such tuning is affected by background loads, target direction, and preparation duration. We found that target distance produced only small variations in reflex gains, but short-latency and long-latency reflexes were strongly tuned according to target direction, in a manner that facilitated the upcoming voluntary reach. This goal-directed tuning of stretch reflex gains was present or enhanced when the preparatory delay was sufficiently long and the homonymous muscle was unloaded.

## Materials and Methods

### Subjects

A total of 16 right-handed, neurologically healthy participants took part in the study; 8 who self-identified as male (mean age, 24.4 ± 4.4 years) and 8 who self-identified as female (mean age, 25.3 ± 6.5 years). All participants were naive as to the specific purposes of the executed task. All were financially compensated for their contribution and gave informed, written consent before participating in the study, per the Declaration of Helsinki. No power calculation was used to predetermine the number of participants to include, but we used a similar or larger number of participants than previous studies (Pruszynski et al., 2009; Yang et al., 2011; Dimitriou et al., 2012; Weiler et al., 2021). Two of the 16 participants did not conform to the experimental manipulations of the study (i.e., presented excessive contraction in shoulder muscles in conditions requiring muscle relaxation/unloading), and their data were therefore not included in further analyses. The current experiments were part of a research program approved by the local Ethics Committee.

### The experimental setup

**Figure 1.**
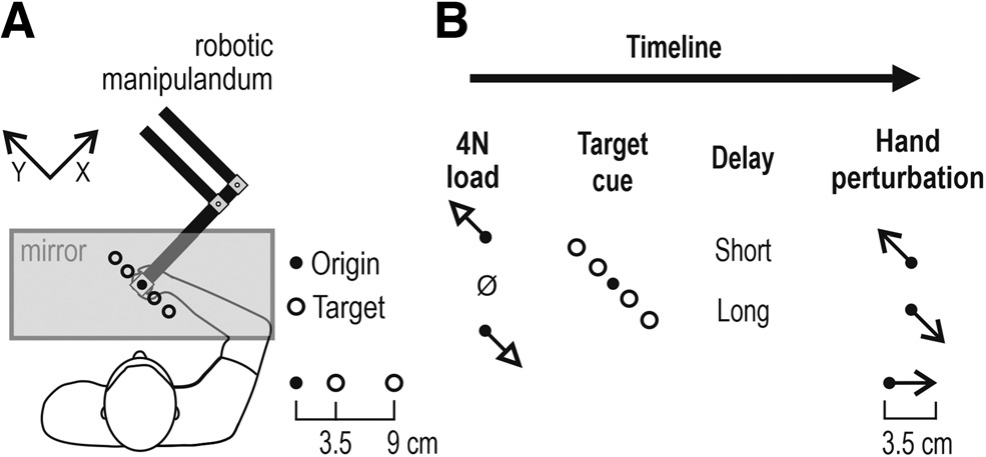
The robotic platform and experimental setup. ***A***, Participants manipulated the position of a robotic handle using the right hand. The right forearm rested on an airsled that allowed frictionless movement. All visual stimuli were projected onto a one-way mirror. The participants could not view their hand or the robotic handle, and the position of the hand was represented by a visual cursor in the plane of movement. The potential visual targets were placed along the *y*-axis (defined as illustrated). Specifically, two near and two far targets were used, placed at 3.5 and 9 cm from the origin, respectively. ***B***, The task timeline. Each trial was initiated when the participant kept the hand/cursor immobile at origin. Either no load was applied or a slow-rising 4 N load in either the +Y (upper left direction) or –Y direction (lower right direction) was then applied. Regardless of load, the participants had to keep their hand at origin. A target was then cued by turning red and remained in this state for either a relatively short (250 ms) or long (750 ms) delay period. This preparatory delay was followed by a rapid (150 ms) perturbation of the hand by 3.5 cm in either the +Y or –Y direction. The cursor position was frozen during the 150 ms perturbation. At the end of the perturbation, the red target turned green (Go signal), and the participants were instructed to rapidly complete movement to the target. All trials were block randomized, meaning that the direction of the kinematic perturbation was unpredictable, even after experiencing a specific load, target cue, and delay.

Participants sat upright in a customized adjustable chair in front of the Kinarm robotic platform (Kinarm End-Point Robot, BKIN Technologies). The participants used their right hand to grasp the robotic manipulandum (Fig. 1*A*). The right forearm was placed inside a customized foam structure, resting on an airsled that allowed frictionless movement of the arm in a 2D plane. To ensure a secure mechanical connection, the forearm, foam-cushioned airsled, the Kinarm handle, and the hand were secured using a leather fabric with Velcro attachments. This attachment also fixated the wrist in straight alignment with the forearm throughout the experiment. The robotic platform measured kinematic data regarding the position of the hand, and sensors inside the robotic handle recorded the forces exerted by the participants’ right hand (six-axis force transducer; Mini40-R, ATI Industrial Automation). Position and force data were sampled at 1 kHz.

### Experimental design

Participants viewed the content of a monitor through the reflection of a one-way mirror that prevented a view of the hand. The position of the right hand was represented by a white dot (“cursor”; diameter, 1 cm) in the plane of movement. The origin was represented by a circle of 1.3 cm diameter. Four visual targets in total were placed along the *y*-axis (Fig. 1*A*). Specifically, two targets were placed in the front-and-left direction (+Y), and two in the right-and-back direction (–Y). The size of all targets was the same (diameter, 2.4 cm). The center-to-center distance between the origin and each “near” target was 3.5 cm, and there was 9 cm between the origin and each “far” target (Fig. 1*A*). All potential targets were continuously displayed as orange circle outlines.

The participant begun each trial by moving the cursor/hand inside the origin circle. There, they had to remain immobile for a random wait period ranging from 1 to 1.5 s. At this point, either no load was applied, or controlled forces could then be applied in the form of a slow-rising 4 N load in either the +Y or –Y direction (rise time, 800 ms; hold, 1200 ms). After the hold phase, one of the four targets then became a red filled circle, representing the target cue. The participant had to remain at the origin for a preparatory delay period of 250 or 750 ms, also referred to as the relatively “short” and “long” delay, respectively. The two preparatory delays were chosen based on previous results, using a similar setup (Dimitriou, 2018; Papaioannou and Dimitriou, 2021). At the end of the preparatory period, a position-controlled perturbation displaced the hand by 3.5 cm in either the +Y or –Y direction (rise time, 150 ms; no hold). The cursor position was frozen at origin during the 150 ms perturbation. The haptic perturbations were used to produce displacements with approximate bell-shaped velocity profiles. The KINARM robot was able to exert the appropriate stiffness (maximum, ∼40,000 N/m), regardless of load/force conditions, to ensure the desired hand kinematics on every trial. Upon the end of the perturbation, the cued target (red filled circle) turned green (representing the “Go” signal), and the participants were instructed to complete the movement to the target. In other words, the Go signal to reach the target was always given on the end of the brief position perturbation.

**Figure 2.**
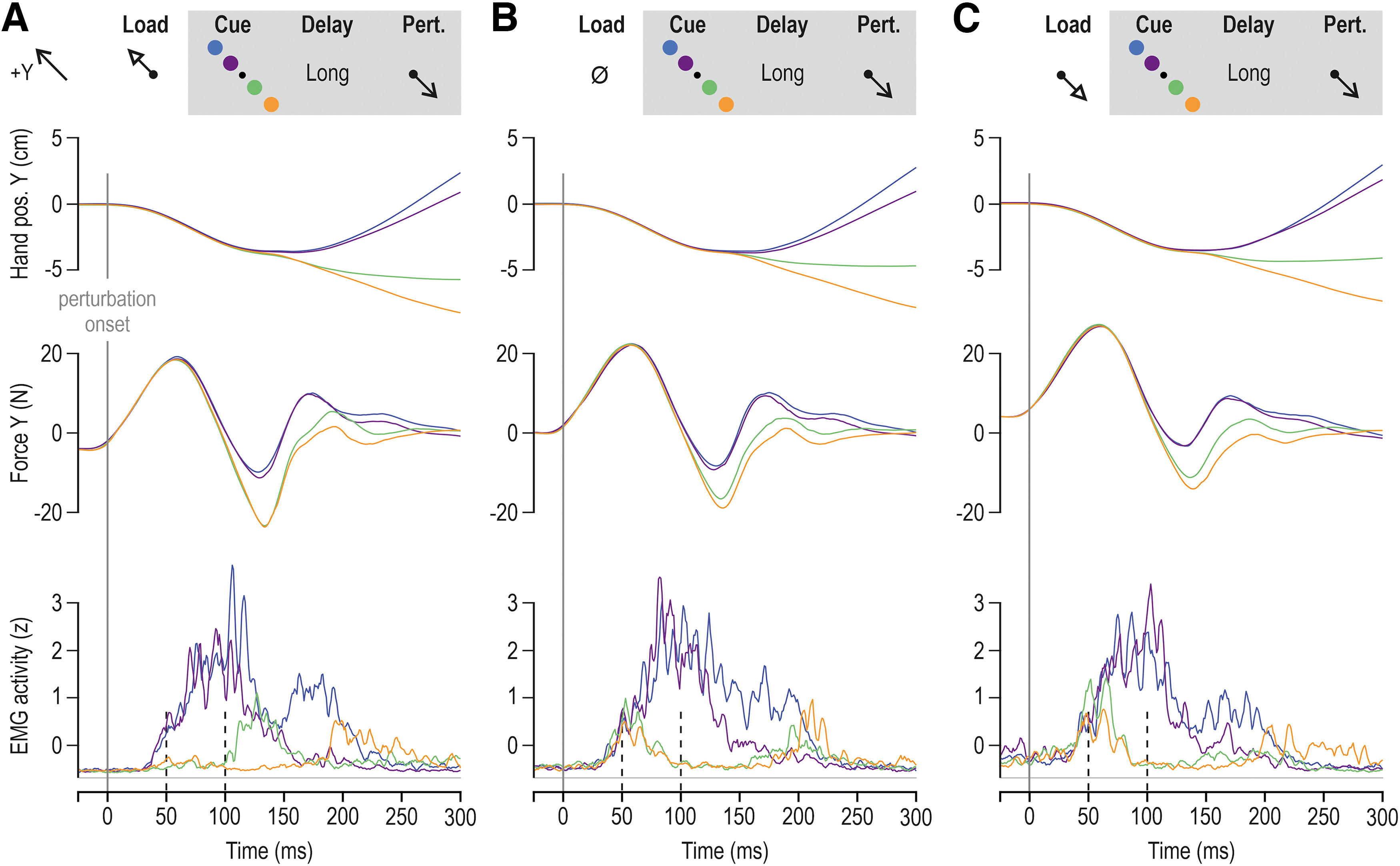
Goal-directed responses from the pectoralis of a single participant in the delayed reaching task. Throughout, blue and purple traces represent trials where participants had to reach for the far and near targets in the +Y direction, respectively (see also schematics). Reaching these targets required shortening of the pectoralis. Orange and green traces represent trials where the participant had to reach the far and near targets in the –Y direction, respectively. These trials require stretch of the pectoralis. All data in this figure involved a long preparatory delay (i.e., 750 ms) and are aligned on perturbation onset (time 0). ***A***, A slow-rising load (“preload”) was first applied in the +Y direction before the visual target cue and subsequent haptic perturbation that stretched the pectoralis. ***B***, As in ***A***, but no slow-rising load was applied. ***C***, As in ***A***, but the slow-rising load was applied in the –Y direction, loading the pectoralis before the visual target cue and subsequent haptic perturbation.

**Figure 3.**
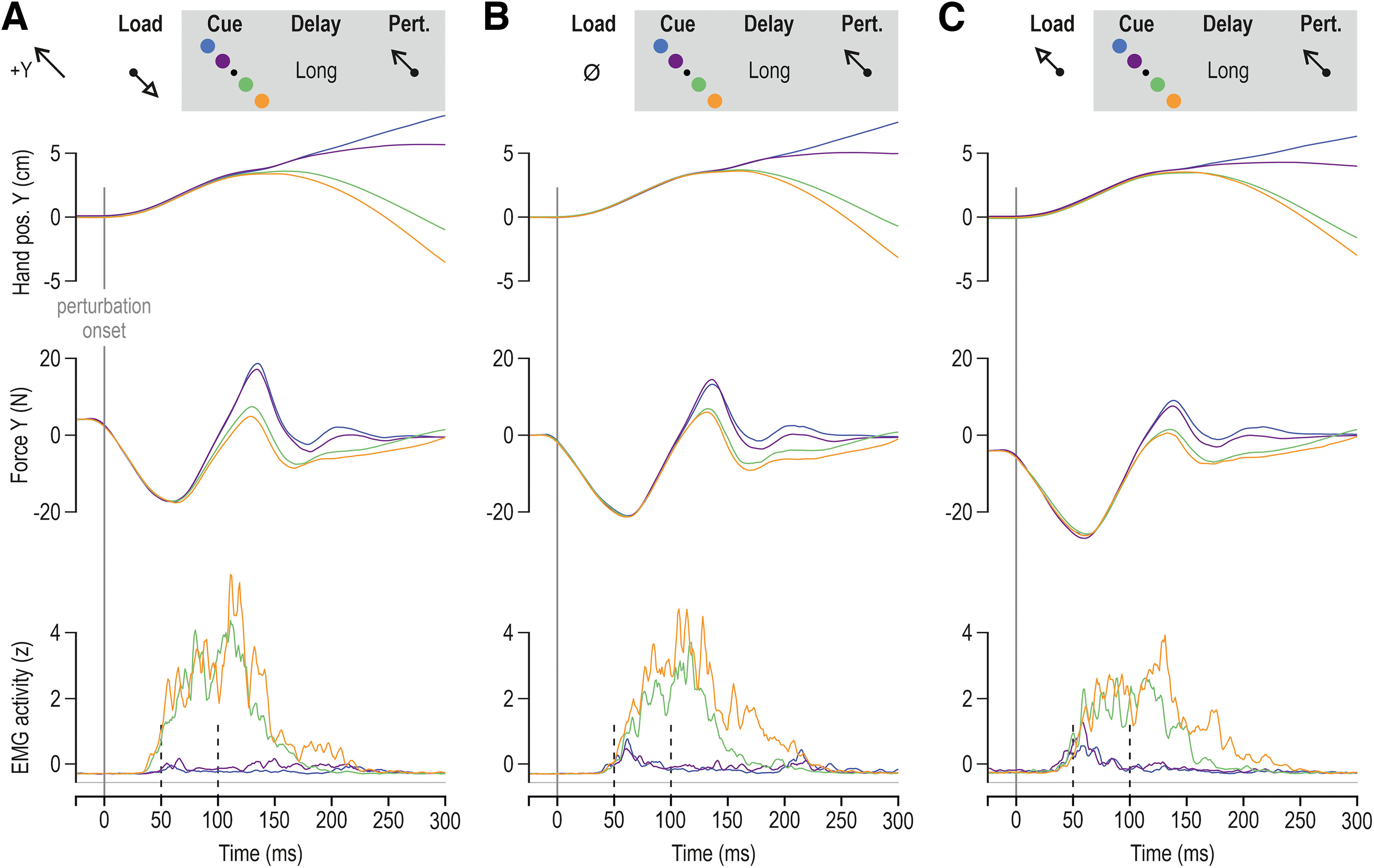
Goal-directed responses from the posterior deltoid of a single participant in the delayed reaching task. Throughout, blue and purple traces represent trials where participants had to reach for the far and near targets in the +Y direction, respectively. Reaching these targets required stretch of the posterior deltoid. Orange and green traces represent trials where the participant had to reach the far and near targets in the –Y direction, respectively. These trials required shortening of the posterior deltoid. All data in this figure involved a long preparatory delay (i.e., 750 ms) and are aligned on perturbation onset (time 0). ***A***, A slow-rising load (preload) was first applied in the –Y direction before the visual target cue and subsequent haptic perturbation that stretched the posterior deltoid. ***B***, As in ***A***, but no slow-rising load was applied. ***C***, As in ***A***, but the slow-rising load was applied in the +Y direction, loading the posterior deltoid before the visual target cue and subsequent haptic perturbation.

Once the cursor/hand reached the target and remained there for 300 ms, the trial ended, and participants were given visual feedback on their performance. The performance metric measured the time from the Go signal until the target was reached. A time faster than 400 ms resulted in a “Too fast” message being shown on the monitor, a time of 400–1400 ms resulted in a “Correct” message, and a time >1400 ms resulted in a “Too Slow” message. The chosen feedback intervals motivated rapid goal-directed behavior (Figs. 2, 3) but also allowed sufficient time for participants to reach the goal regardless if the hand was first perturbed in an incongruent direction. After receiving visual feedback (lasting for 300 ms), the participants returned the cursor inside the origin to start the next trial. After any trial, the participant could move the cursor to the side of the workspace and rest, although breaks were normally encouraged and requested after a substantial number of trials. Breaks normally lasted for <5 min. The experiment consisted of 48 unique trial types: four targets (+Y far, +Y near, –Y far, and –Y near) × three load conditions (4 N in +Y direction, null-load, and 4 N in –Y direction) × two delay durations (250 and 750 ms) × two perturbation directions (+Y and –Y). The participants performed 15 repetitions of each condition (i.e., the total number of trials in each experiment was 720). The trials were presented in a block-randomized order, where one “block” represented a set of 48 unique trials. The delayed-reach task took ∼1.5 h to complete.

### Electromyography

Surface electromyography (EMG) signals were recorded from (1) musculus (m.) brachioradialis, (2) m. biceps brachii, (3) m. triceps brachii caput laterale, (4) m. triceps brachii caput longum, (5) m. deltoideus pars anterior (6) m. deltoideus pars posterior, and (7) m. pectoralis major. However, in line with previous findings, it was the latter three muscles (i.e., shoulder actuators: pectoralis, anterior, and posterior deltoid) that were primarily engaged in the delayed reach task of our setup, hence only these three muscles were used for statistical analyses. We used surface EMG electrodes (Bagnoli DE-2.1, Delsys) that have contact dimensions 10.0 × 1.0 mm with 10 mm interelectrode spacing. Before attaching the electrodes, the skin was cleaned using alcohol swabs. The electrodes were coated with conductive gel and placed on the peak of the belly of the studied muscles in the direction of the muscle fibers. All electrodes were attached with double-sided tape and further secured using surgical tape. One ground electrode (Dermatrode HE-R Reference Electrode type 00200–3400, American Imex), with a diameter of 5.08 cm, was placed on the processus spinosus of the C7 region. The EMG signals were analog bandpass filtered through the EMG system (20-450 Hz) and sampled at 1 kHz.

### Data preprocessing

EMG data were high-pass filtered using a fifth-order, zero phase-lag Butterworth filter with a 30 Hz cutoff and then rectified. For each trial, the onset of movement was defined as the point where velocity first reached 5% of peak velocity during movement. Normalization was applied to allow EMG data from different muscles and participants to be combined and/or compared. The raw data were normalized (*z*-transformed) using a procedure that has been described in more detail previously (Dimitriou, 2014, 2016). Briefly, this involves concatenating all EMG data (here, from all 15 blocks) and calculating a grand mean and grand SD for each muscle separately. Normalized EMG data for each muscle are obtained by subtracting the grand mean and dividing it by the grand SD. An alternative normalization strategy was applied to further evaluate the preperturbation period. For each trial type and muscle/participant, we produced the average (mean) unnormalized EMG trace across repetitions (aligned on perturbation onset). For each muscle, the maximal EMG value across all averaged traces (i.e., across trial types) observed anytime 50 ms after perturbation onset was used to normalize all data for that muscle. In other words, we have used this maximal averaged value as a proxy for maximal voluntary contraction and normalized the EMG data as a proportion (percentage) of this value.

The first five blocks of trials were viewed as familiarization trials and were not included in the analyses. The focus of this study was on stretch reflex responses; therefore, we only analyzed data from stretching muscles. That is, we analyzed specific combinations of muscle and perturbation direction. To simplify analyses of individual muscles, the median EMG signal of each muscle was generated for each trial type (i.e., average for each load, perturbation, preparatory delay, target direction, and distance) for each participant. Data preprocessing was performed using MATLAB (version 2020b; MathWorks). For plotting purposes only, the EMG signals were smoothed using a 5 ms moving window.

### Statistics

**Figure 4.**
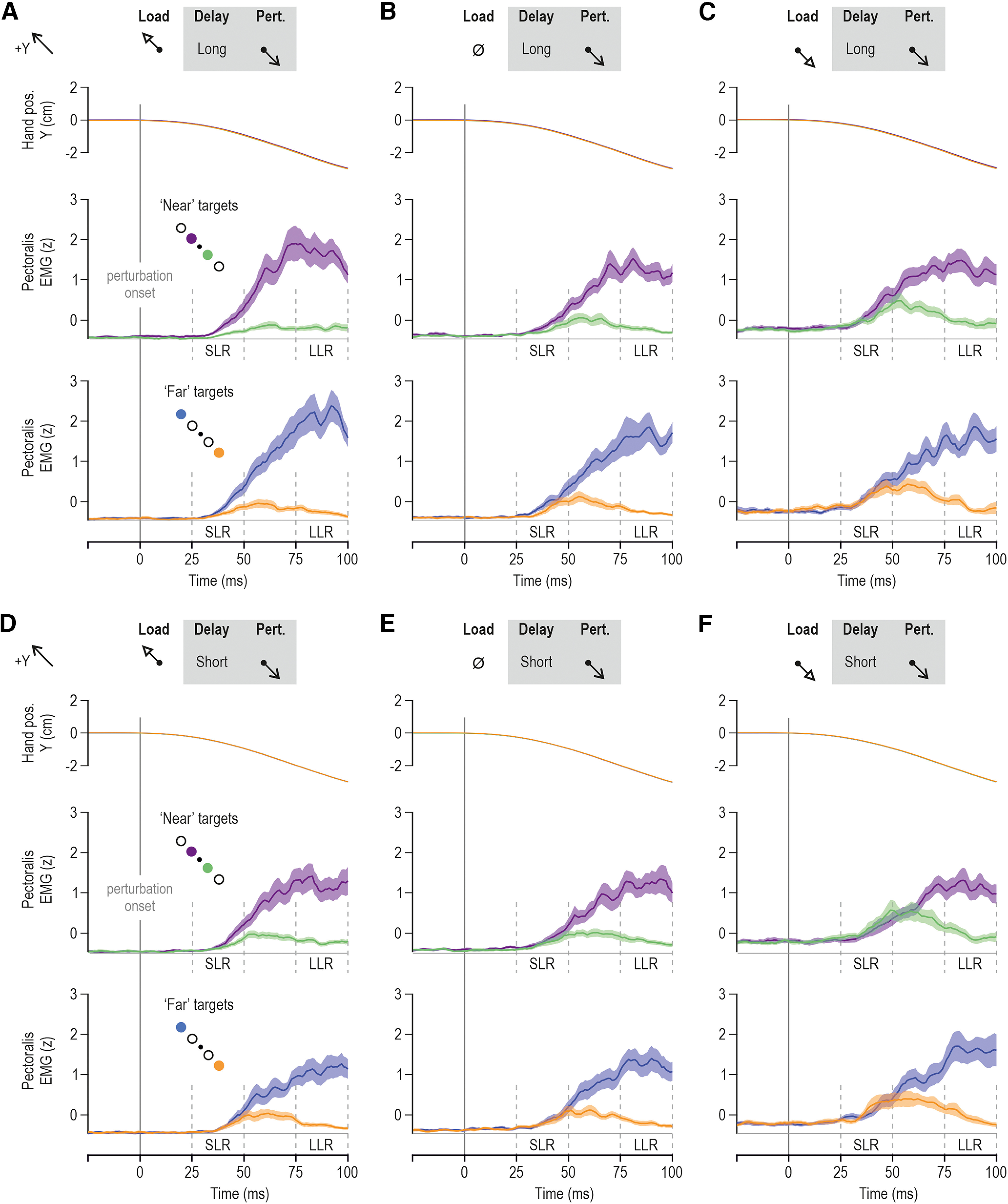
Goal-, load-, and delay-dependent stretch reflex responses of the pectoralis. Color coding is as in previous figures. ***A***, The top panel represents mean hand position across participants (*N* = 14) for all trials where the pectoralis muscle was unloaded before being stretched by the perturbation, following a long preparatory delay (750 ms). The middle row displays mean pectoralis EMG activity across participants for the subset of trials where one or the other near targets were cued for a long delay before pectoralis stretch; the bottom panel represents the equivalent for far targets. ***B***, As in ***A***, but representing the no-load trials. ***C***, As in ***A***, but representing trials where the pectoralis was loaded before the stretch perturbation. ***D–F***, As in ***A–C***, but representing trials where the preparatory delay was short. See also schematics. Throughout, color shading represents ±1 SEM.

**Figure 5.**
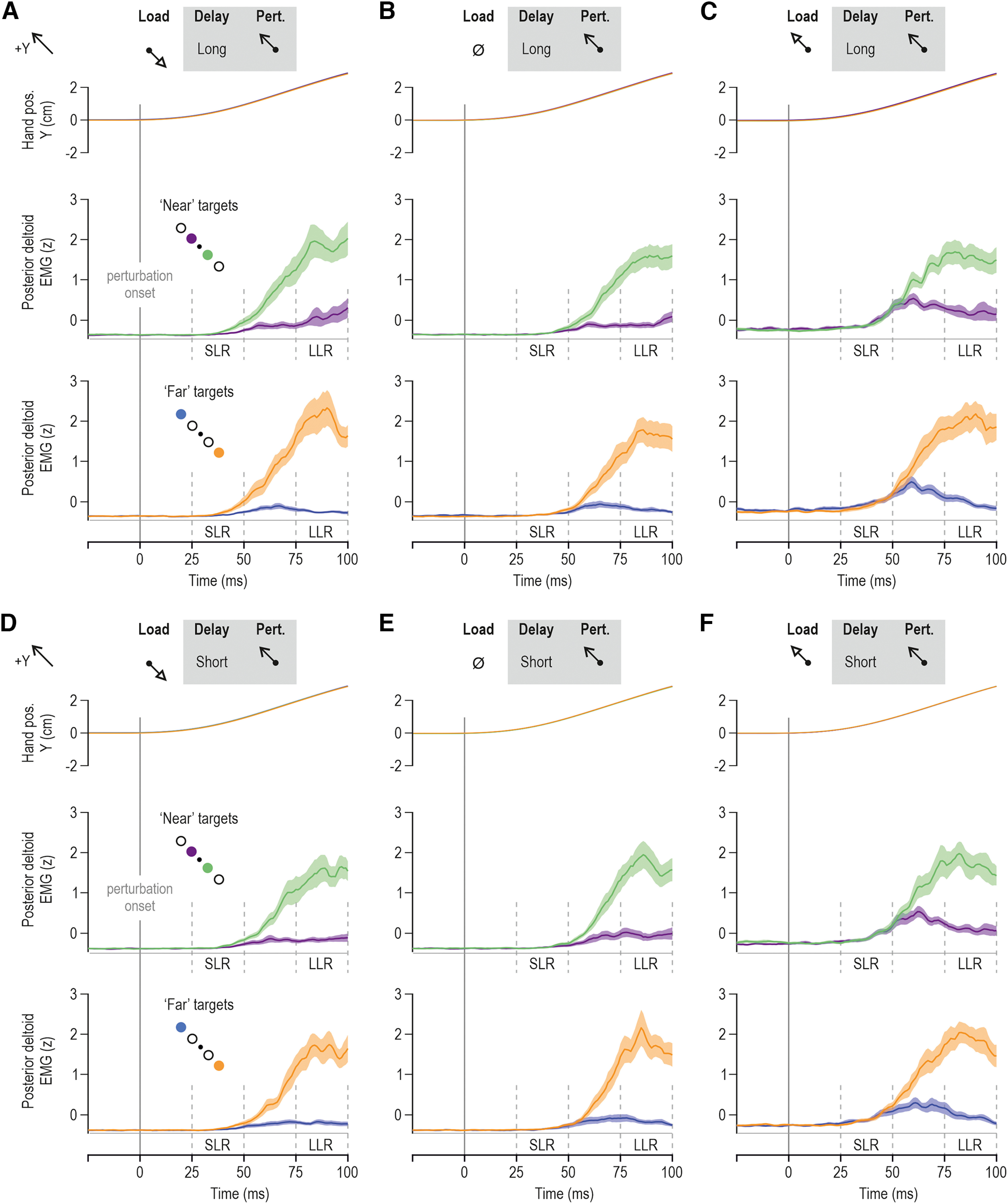
Goal-, load-, and delay-dependent stretch reflex responses of the posterior deltoid. Color coding is as in previous figures. ***A***, Top, Mean hand position across participants (*N* = 14) for all trials where the posterior deltoid was unloaded before being stretched by the perturbation, following a long preparatory delay (750 ms). Middle, The mean posterior deltoid EMG activity across participants for the subset of trials where one or the other near targets were cued for a long delay before posterior deltoid stretch. Bottom, The equivalent for far targets. ***B***, As in ***A***, but representing the no-load trials. ***C***, As in ***A***, but representing trials where the posterior deltoid was loaded before the stretch perturbation. ***D–F***, As in ***A–C***, but representing trials where the preparatory delay was short. See also schematics. Throughout, color shading represents ±1 SEM.

**Figure 6.**
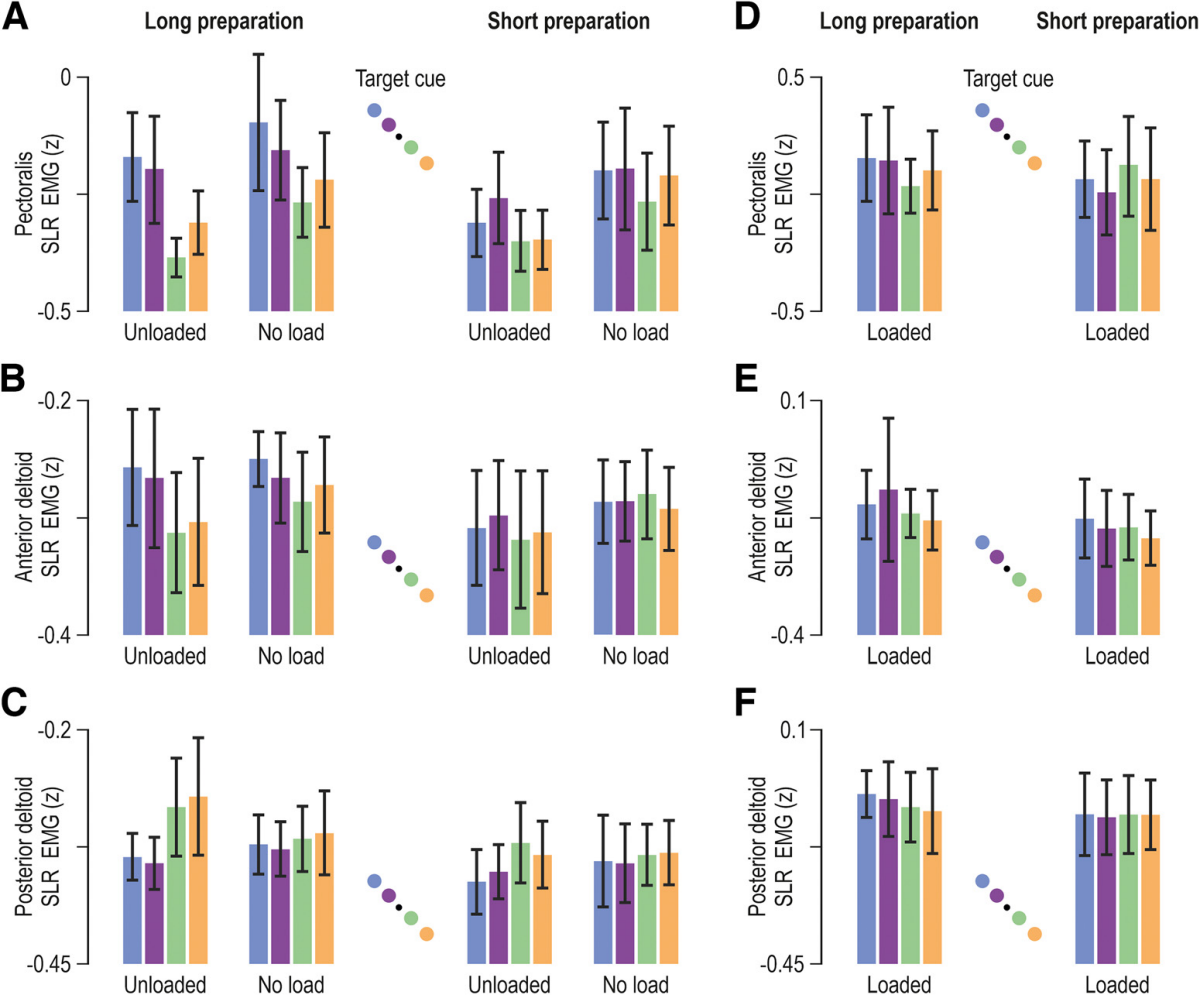
Goal-directed modulation of short-latency reflex gains. ***A***, The colored bars represent mean pectoralis SLR EMG (*z*) across participants (*N* = 14), and vertical lines represent 95% confidence intervals. Color coding is as in previous figures (see also schematics). Data originate from trials where the homonymous muscle was unloaded and when there was no preload, as indicated. An ANOVA confirmed a consistent effect of target direction on pectoralis SLR gains when the preparatory delay was long (left columns). That is, SLR gains are relatively suppressed when allowed long enough time to prepare reaching a target along the direction of homonymous muscle stretch (green/orange). ***B***, As in ***A***, but representing the anterior deltoid muscle. Similar SLR modulation patterns were observed as for the pectoralis (see Results section for more details). ***C***, As in ***A***, but representing the posterior deltoid. ***D–F***, As in ***A–C*** but representing trials where the homonymous muscle was loaded.

Statistical analyses were performed on the *z*-normalized EMG data [available at Mendeley Data, V1 (doi: 10.17632/hnfp5yrght.1)]. To check normality, Shapiro–Wilk tests for samples with <50 datapoints and Lilliefors tests for larger samples were used. For each muscle, the data used for statistical analyses were the averages across the preperturbation epoch (i.e., the period starting 20 ms before perturbation onset), the SLR epoch (25–50 ms postperturbation onset), and the LLR epoch (76–100 ms postperturbation onset). To analyze preperturbation and reflex EMG responses, a repeated-measures ANOVA of the design 2 (preparatory delay) × 3 (load) × 2 (target direction) × 2 (target distance) was used. For the SLR, in particular, the preloaded (“loaded”) muscle condition was analyzed separately (i.e., 2 delay × 2 distance × 2 direction), as it is well known that automatic gain scaling because of muscle preloading tends to saturate the SLR, preventing its goal-directed modulation, as occurred in our study (Figs. 2-6; also see Results). Throughout, *post hoc* analyses were performed using Tukey’s HSD test.

To estimate the onset of SLR reflex modulation, we used the receiver operating characteristic (ROC) technique (Green and Swets, 1966). An ROC area of 1 and 0 signifies perfect discrimination while an ROC area of 0.5 signifies a discrimination performance equal to chance. For this type of analysis, we only used data from trials where the preparatory delay was long. Specifically, the EMG curves of targets in the direction of homonymous muscle stretch were contrasted to EMG responses observed in trials where the cued target was in the direction of muscle shortening. The obtained averages across target distance were together viewed as representing the reflex modulation in the population sample. Discrimination was viewed as significant when the ROC area remained >0.75 for five consecutive samples (Corneil et al., 2004). To assess the reflex modulation onset for each participant separately, the same type of procedure was performed using individual EMG responses across trials. All SLR modulation onsets were confirmed by visual inspection, to eliminate the risk of false positives. Data tabulation was performed using MATLAB (version R2020b; MathWorks), and statistical analyses were performed using STATISTICA (StatSoft).

## Results

Participants were asked to hold their right hand within a starting position while one of three different loads was applied (−4, 0, or 4 N). One of four targets was then cued (turned red), and after either a short or long delay the limb was perturbed by 3.5 cm in one of two directions (+Y or –Y; Fig. 1*A*). After the 150 ms perturbation, the target color was changed from red to green, indicating that the participants should move the hand to this target. Here we examine how the target location (direction and distance), background load, and delay between the presentation of the target and the perturbation effected the reflex responses before the movement of the participants.

Representative data from single participants are presented in Figures 2 and 3, for the pectoralis major and posterior deltoid, respectively. Visual inspection of both figures points to differences in EMG during the SLR epoch as a function of target cue, particularly when the muscles were unloaded (Figs. 2*A*, 3*A*). Specifically, the SLR appears to be relatively suppressed when the cued target is in the direction of muscle stretching (e.g., blue/purple vs green/orange for the pectoralis; Fig. 2*A*). Relative suppression of stretch reflexes—and therefore of muscle stiffness—would facilitate the subsequent reaching movement. Clear goal-directed differences in the LLR epoch can be seen across all load conditions (Figs. 2, 3). As elaborated in the Materials and Methods section, the modulation of stretch reflex responses was analyzed using averaged data across participants. Figure 4 shows the averaged hand position and pectoralis EMG activity aligned on perturbation onset (time 0), whereas Figure 5 displays equivalent responses from the posterior deltoid.

### The preperturbation epoch

To confirm the lack of goal-directed differences in the preperturbation epoch (i.e., the 20 ms period before perturbation onset), an ANOVA of the design 2 (preparatory delay) × 3 (load) × 2 (target distance) × 2 (target direction) was performed using averaged EMG data over this epoch. As expected, for all investigated muscles, there was a significant main effect of load condition on preperturbation EMG. Specifically, for the pectoralis, ANOVA indicated a significant main effect of load (*F*_(2,26)_ = 41.5, *p* < 10^−5^, and η^2^_p_ = 0.76), whereas all other main and interaction effects were not significant (all *p* > 0.065). The Tukey’s HSD test showed that preperturbation EMG was significantly higher only in the loaded condition (vs “no-load” and “unloaded” conditions, all *p* < 0.0002; Fig. 4*C*,*F*). As mentioned above, the plots point to a particularly clear effect of target direction on reflex responses when the homonymous muscle is unloaded (Fig. 4*A*); any equivalent differences in preperturbation EMG in the unloaded condition could possibly account for the enhanced SLR effects because of gain scaling. However, a planned comparison test indicated no impact of target direction on preperturbation EMG when the pectoralis was unloaded (*p* = 0.63). The same results were obtained for the anterior deltoid. That is, the ANOVA produced a significant main effect of load (*F*_(2,26)_ = 5.3, *p* = 0.012, and η^2^_p_ = 0.3), whereas all other main and interaction effects were not significant (all *p* > 0.11). The Tukey’s HSD test indicated significantly higher preperturbation EMG only when the muscle was loaded (vs unloaded, *p* = 0.016; vs no load, *p* = 0.035). A planned comparison also indicated no specific effect of target direction on preperturbation EMG when the anterior deltoid was unloaded (*p* = 0.36).

For the posterior deltoid, an ANOVA again showed a main effect of load (*F*_(2,26)_ = 57.8, *p* < 10^−5^, and η^2^_p_ = 0.82) as with the other two muscles (i.e., higher EMG in loaded condition, all *p* < 0.0002). But there was also a main effect of preparatory delay, with generally higher preperturbation EMG observed when the preparatory delay was long (*F*_(1,13)_ = 18.4, *p* = 0.0009, and η^2^_p_ = 0.59). ANOVA also indicated significant interaction effects, such as among delay, load condition, and target direction (*F*_(2,26)_ = 6.1, *p* = 0.007, and η^2^_p_ = 0.32). However, as can be appreciated by visually inspecting the preperturbation epochs in Figure 5, differences in posterior deltoid EMG as a function of target parameters were apparent only when the muscle was loaded. Indeed, performing the ANOVAs without including the loaded condition eliminated all effects involving target parameters (all *p* > 0.05); only a significant main effect of preparatory delay remained (*F*_(1,13)_ = 14.8, *p* = 0.002, and η^2^_p_ = 0.53), indicating higher preperturbation activity in the posterior deltoid following a long delay, regardless of target direction or distance. Furthermore, a planned comparison test indicated no significant effect of target direction on preperturbation EMG when the posterior deltoid was unloaded (*p* = 0.59). Importantly, there are no consistent target-dependent differences in the preperturbation epoch for the posterior deltoid, anterior deltoid, and pectoralis muscles of the dominant upper limb.

Similarly, when the alternative normalization procedure was used, there was no significant impact of target direction on the preperturbation EMG of the unloaded pectoralis (*p* = 0.32) or on the preperturbation activity of the unloaded anterior and posterior deltoids (*p* = 0.55 and *p* = 0.5, respectively). In contrast to the case of the unloaded pectoralis, where target direction impacted SLRs following either a long or short delay (Fig. 6*A*), target direction modulated the SLR of the unloaded posterior and anterior deltoids only after a long preparatory delay (Fig. 6*B*,*C*). However, we again found no impact of target direction on preperturbation activity when the above planned comparisons were conducted only for trials where the preparatory delay was long, with *p* = 0.6 for the anterior deltoid and *p* = 0.62 for the posterior deltoid.

### The SLR epoch

As the main aim of this study was to further investigate the preparatory modulation of stretch reflex gains, we focus the rest of the analysis on the specific responses during muscle stretch. Specifically, all data originate from trials where the hand was perturbed along the direction of targets associated with stretch of the homonymous muscle, regardless of voluntary movement intent. Moreover, it is well known that automatic gain scaling because of preloading of the muscle tends to saturate the SLR, limiting its goal-directed modulation (Figs. 2-5). Therefore, SLRs from the preloaded (loaded) homonymous muscles were analyzed separately, using an ANOVA design of 2 (preparatory delay) × 2 (target distance) × 2 (target direction). Analysis of SLRs associated with the no-load and unloaded conditions was performed using an ANOVA design of 2 (preparatory delay) × 2 (load) × 2 (target distance) × 2 (target direction). The following text focuses on and first describes the results involving the unloaded and no-load conditions. Throughout, differences in the stretch reflex responses—at all latencies—represent differences in stretch reflex gains, as both the initial position and kinematic perturbation of the hand are matched across relevant experimental conditions.

For the pectoralis SLR (Fig. 6*A*), ANOVA indicated a significant main effect of target direction (*F*_(1,13)_ = 12.1, *p* = 0.004, and η^2^_p_ = 0.48), with stronger responses observed when the target cue was placed along the direction of pectoralis shortening (i.e., the +Y direction). There was also a main effect of preparatory delay (*F*_(1,13)_ = 5.3, *p* = 0.04, and η^2^_p_ = 0.29) and an interaction effect between preparatory delay and target direction (*F*_(1,13)_ = 8.5, *p* = 0.012, and η^2^_p_ = 0.4). The Tukey’s HSD test indicated that the impact of target direction (blue/purple > orange/green; Fig. 6*A*) was significant only following a long preparatory delay (all *p* < 0.008: mean blue/purple, −0.18 ± 0.09 95% CI; vs mean orange/green, −0.29 ± 0.09 95% CI). However, a planned comparison analysis revealed an additional significant effect of target direction on the SLRs of the unloaded muscle following a short preparatory delay (i.e., purple > green, *p* = 0.036; mean purple, −0.27 ± 0.09 95% CI vs mean green, −0.35 ± 0.08 95% CI); but, this effect was less pronounced than the equivalent following a long delay (Fig. 4, compare *A*, *D*). There was also a main effect of target distance on pectoralis SLR, with stronger overall responses evident for far versus near targets (*F*_(1,13)_ = 9.2, *p* = 0.0094, and η^2^_p_ = 0.42). The interaction effect between preparatory delay and target distance failed to reach significance (*F*_(1,13)_ = 4.4, *p* = 0.056, and η^2^_p_ = 0.25). Interestingly, there was also a main effect of load condition on pectoralis SLR, with stronger EMG responses in the no-load versus unloaded condition (*F*_(1,13)_ = 8, *p* = 0.014, and η^2^_p_ = 0.38).

Similar results were obtained with regard to the SLRs of the anterior deltoid (Fig. 6*B*). Specifically, an ANOVA indicated a significant main effect of target direction (*F*_(1,13)_ = 11.6, *p* = 0.005, and η^2^_p_ = 0.47), with stronger SLRs when the target cue was placed along the direction of muscle shortening. The ANOVA also revealed a main effect of preparatory delay on anterior deltoid SLR, with the long delay associated with higher responses (*F*_(1,13)_ = 8.6, *p* = 0.012, and η^2^_p_ = 0.4). For the same muscle, there was also a significant interaction effect between preparatory delay and target direction (*F*_(1,13)_ = 5.2, *p* = 0.04, and η^2^_p_ = 0.27). The Tukey’s HSD test indicated significantly higher SLRs in the long delay condition when the target cue was in the direction associated with shortening of the anterior deltoid (all *p* < 0.008). However, there was no main effect or interaction effect involving target distance for this muscle (all *p* > 0.5). Overall, for both the anterior deltoid and pectoralis, there was a clear goal-directed modulation of SLR gains in cases where a long enough time (>250 ms) was allowed for preparing a stretch of the homonymous muscle (Fig. 6*A*,*B*, far left columns).

With regard to the SLRs of the posterior deltoid (Fig. 6*C*), an ANOVA indicated both a main effect of preparatory delay (*F*_(1,13)_ = 12.7, *p* = 0.0034, and η^2^_p_ = 0.49) and target direction (*F*_(1,13)_ = 7.2, *p* = 0.019, and η^2^_p_ = 0.36), with higher SLR EMG for longer preparatory delays and for target cues presented along the direction of muscle shortening (i.e., –Y direction for the posterior deltoid). There was also an interaction effect between load and target direction (*F*_(1,13)_ = 5.4, *p* = 0.037, and η^2^_p_ = 0.29), with *post hoc* analyses indicating a significant impact of target direction on posterior deltoid SLR only in the unloaded condition (*p* = 0.0048: mean orange/green, −0.28 ± 0.05 95% CI; vs mean blue/purple, −0.34 ± 0.02 95% CI). For this muscle, there was also a significant interaction effect between preparatory delay and target distance (*F*_(1,13)_ = 8.4, *p* = 0.012, and η^2^_p_ = 0.39), although the Tukey’s HSD test indicated no differential impact of target distance as a function of preparatory delay (Fisher’s test, on the other hand, indicated a significant impact of target distance when the delay was long; *p* = 0.026). Overall, we again find a clear target-related modulation of the SLR in the unloaded condition when sufficient preparation time is provided (Fig. 6*C*, far left column).

Figure 6*D–F* displays SLRs across participants when the homonymous muscle was loaded. ANOVA analyses (2 delay × 2 target distance × 2 target direction) indicated no significant main effect (or interaction effect) of target location on the SLRs of the three analyzed muscles (all *p* > 0.28 for anterior deltoid; all *p* > 0.1 for pectoralis; all *p* > 0.39 for posterior deltoid). For the loaded anterior deltoid alone (Fig. 6*E*), there was a significant main effect of preparatory delay on SLRs (long>short; *F*_(1,13)_ = 7.95, *p* = 0.014, and η^2^_p_ = 0.38), whereas no such effect was observed for the posterior deltoid and pectoralis (*p* = 0.15 and *p* = 0.14, respectively). The above analyses confirm what can be visually appreciated by inspecting SLRs in Figures 2-6: loading the homonymous muscle substantially for the purposes of postural maintenance tends to saturate the SLR, thwarting its preparatory modulation according to task goals.

In summary, across the three analyzed muscles, ANOVA yielded no consistent effect of target distance on SLRs. That is, an effect of target distance on SLR (far > near targets) was evident only for the pectoralis, if the muscle was not first loaded. In contrast, there was a consistent effect of target direction. Specifically, all muscles produced goal-directed SLRs, in the sense of displaying relatively weaker/stronger reflex gains, when preparing to reach targets requiring stretch/shortening of the homonymous muscle. These response patterns were present or clearer when the preparation time was sufficiently long and the muscles were unloaded (i.e., when a background load was first applied in the direction of muscle action; Fig. 6, far left columns).

**Figure 7.**
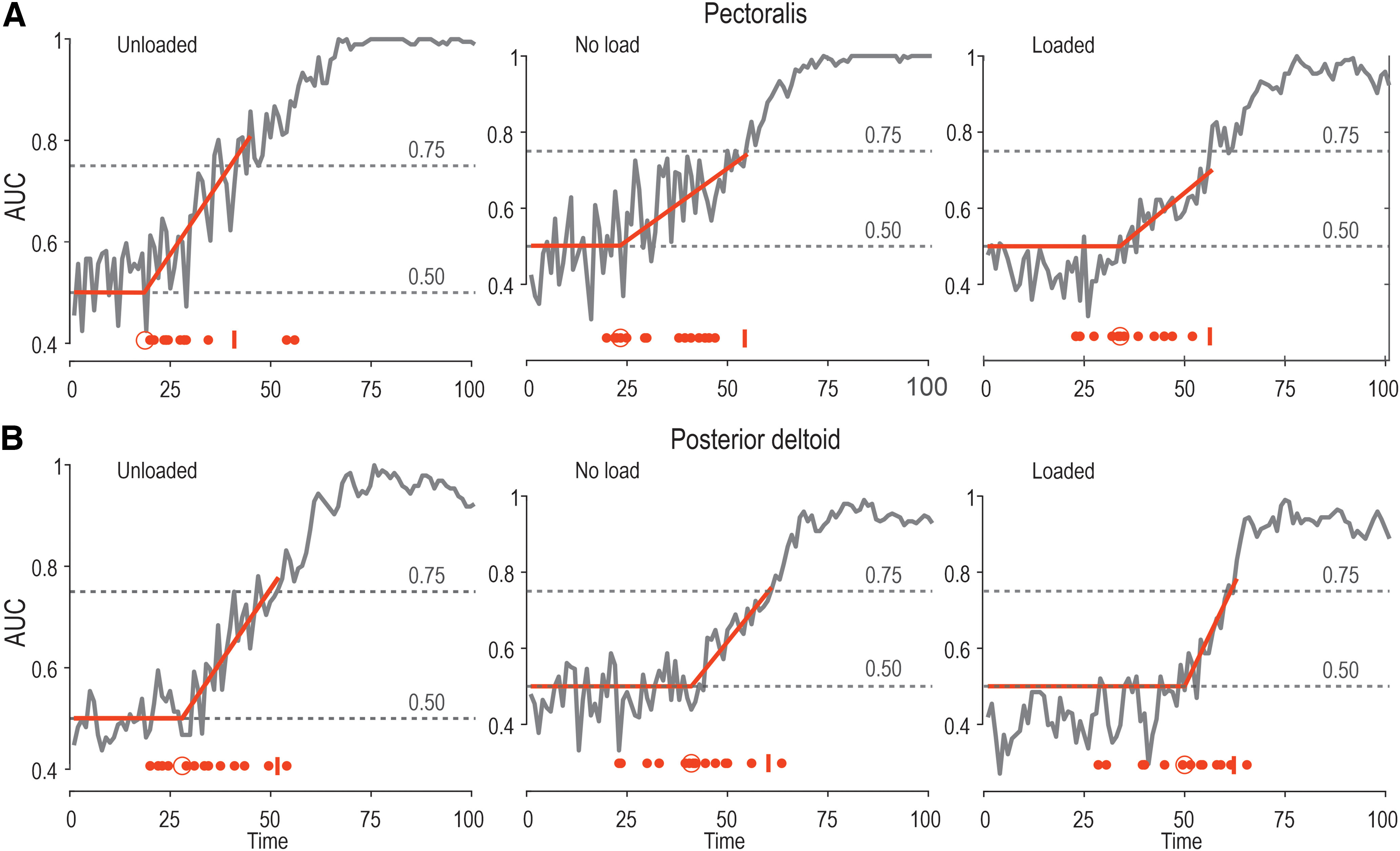
The time onset of SLR modulation. ***A***, The gray curve in each panel represents the area under the ROC, pertaining to pectoralis SLR modulation as a function of target direction, after experiencing one of the three load conditions (unloaded, no load, and loaded) and a long preparatory delay (see Materials and Methods and Results for more details). Specifically, vertical axes represent the probability that an ideal observer could discriminate between the EMG difference curves. Each solid red line represents a dog leg fit, which was applied to determine the onset of significant SLR modulation as a function of target direction (see also larger red circle at the bottom of each panel). The small red vertical line at the bottom of each panel represents the time point when the ROC area remained >0.75 for five consecutive time points (i.e., five consecutive ms). The smaller red dots represent the ROC result for each individual participant. ***B***, As in ***A***, but representing the posterior deltoid.

To further characterize the goal-directed modulation of the SLR, ROC analysis was applied. Since ANOVA indicated no consistent effect of target distance on SLRs, the data were collapsed across target distance, to concentrate on the impact of target direction. The relevant difference signals were created by contrasting the EMG curve observed when reaching for targets requiring muscle stretch versus when reaching for targets requiring muscle shortening. These were used to determine the time point at which the signals could be discriminated by an ideal observer. For the unloaded pectoralis (Fig. 7*A*), dog leg fits indicated deviance at 19 ms; for the no-load condition, at 23 ms; and for the loaded condition, at 34 ms. For the unloaded posterior deltoid (Fig. 7*B*), this occurred at 28 ms; for the no-load condition, at 41 ms; and for the loaded condition, at 50 ms. The onset times were also calculated for each participant individually (Fig. 7*A*,*B*, small red circles). The time point at which the ROC was >0.75 was also identified. For the unloaded pectoralis (Fig. 7*A*), this occurred at 45 ms; for the no-load condition, at 55 ms; and for the loaded condition, at 57 ms. For the unloaded posterior deltoid (Fig. 7*B*), this occurred at 52 ms; for the no-load condition, at 61 ms; and for the loaded condition, at 63 ms (Fig. 7*B*, red vertical lines).

### The LLR epoch

**Figure 8.**
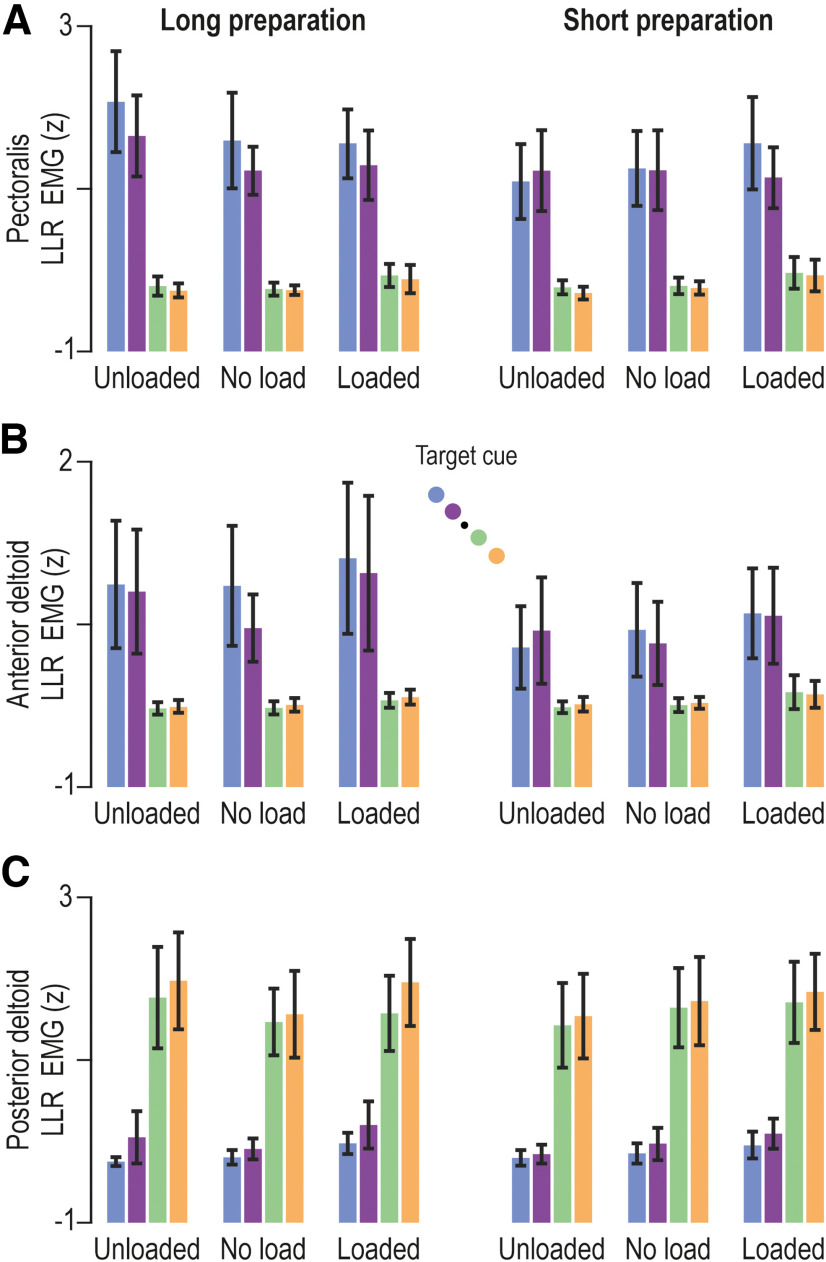
Goal-directed modulation of long-latency reflex gains. ***A***, The colored bars represent the mean pectoralis LLR EMG (*z*) across participants (*N* = 14), and vertical lines represent 95% confidence intervals. Color coding as in previous figures (see also schematic in ***B***). ANOVAs indicated a significant impact of target direction on pectoralis LLR gains but, in contrast with the SLR results (Fig. 6*A*), the impact of target direction was significant on LLR gains also when the preparatory delay was short. However, there was also an effect of preparatory delay on LLR gains. ***B***, As in ***A***, but representing the anterior deltoid muscle. Similar LLR modulation patterns were observed as for the pectoralis. ***C***, As in ***A***, but representing the posterior deltoid.

The modulation of LLR gains largely paralleled the effects seen at the SLR epoch, with an equivalently prominent impact of target direction. As mentioned above, all data in this section refer to stretch of the homonymous muscle (i.e., all data originate from trials where the hand was perturbed along the direction of targets associated with stretch of the homonymous muscle, regardless of voluntary movement intent). Moreover, for the purposes of the LLR analyses, all load conditions were examined together. That is, because goal-directed modulation of LLR gains is known to be robust against automatic gain scaling, here we used the full ANOVA design of 2 (preparatory delay) × 3 (load) × 2 (target distance) × 2 (target direction). As expected (Fig. 8), LLRs of all three muscles were strongly modulated as a function of target direction, with higher stretch reflex gains evident when preparing to reach targets associated with shortening of the homonymous muscle.

Specifically, for the pectoralis muscle (Fig. 8*A*), the ANOVA indicated a significant main effect of target direction on LLRs (*F*_(1,13)_ = 77.2, *p* < 10^−5^, and η^2^_p_ = 0.86), a significant main effect of preparatory delay (*F*_(1,13)_ = 5.8, *p* = 0.032, and η^2^_p_ = 0.31), and a significant interaction between preparatory delay and target direction (*F*_(1,13)_ = 8.4, *p* = 0.012, and η^2^_p_ = 0.4). The Tukey’s HSD test indicated that all comparisons involving target direction and delay were significantly different (all *p* < 0.009), with the exception of no significant effect of delay duration when preparing to reach a target in the direction of pectoralis lengthening (*p* = 0.99; Fig. 8*A*, green and orange bars). In other words, delay duration played no role when the imposed pectoralis stretch was congruent with the goal of the intended movement. Instead, experiencing a long delay was associated with even stronger LLRs when the hand was subsequently perturbed in the direction opposite to that of the intended reach (Fig. 8*A*, blue and purple bars). Furthermore, there is a significant interaction effect between preparatory delay and load (*F*_(2,26)_ = 11.4, *p* = 0.0003, and η^2^_p_ = 0.47), and a further interaction effect among delay, load, and target direction (*F*_(2,26)_ = 6.1, *p* = 0.007, and η^2^_p_ = 0.32; there is no main effect of load: *F*_(2,26)_ = 1.8, *p* = 0.36). *Post hoc* analyses revealed that LLR EMG was highest when the pectoralis was unloaded, and the hand was perturbed in an incongruent direction following a long preparatory delay (Fig. 8*A*, left-most blue and purple bars; all *p* < 0.0017). Hence, paralleling the SLR tuning of this muscle, goal-directed tuning of LLR was most prevalent when the unloaded pectoralis was perturbed/stretched following a long enough preparatory delay (>250 ms). There is a significant but weak effect of target direction on LLRs (*F*_(1,13)_ = 5, *p* = 0.043, and η^2^_p_ = 0.28) and a strong interaction effect between target distance and target direction (*F*_(1,13)_ = 10.2, *p* = 0.007, and η^2^_p_ = 0.44). The Tukey’s HSD test indicated significantly higher LLR EMG for far versus near targets only when these were placed along the pectoralis shortening direction (i.e., blue vs purple, *p* = 0.01; Fig. 8*A*, green vs orange targets, *p* = 0.91).

Similar results were obtained for the anterior deltoid (Fig. 8*B*). An ANOVA indicated a significant main effect of target direction on LLRs (*F*_(1,13)_ = 19.2, *p* = 0.0008, and η^2^_p_ = 0.6), a significant main effect of preparatory delay (*F*_(1,13)_ = 12.3, *p* = 0.004, and η^2^_p_ = 0.49), and an interaction effect between preparatory delay and target direction (*F*_(1,13)_ = 13.9, *p* = 0.0026, and η^2^_p_ = 0.52). The Tukey’s HSD test showed that all comparisons involving target direction and delay were significantly different (all *p* < 0.0016), with the exception of no significant effect of delay duration when preparing to reach a target in the direction of pectoralis lengthening (*p* = 0.98; Fig. 8*B*, green and orange bars). Like the case of the pectoralis, preparing to voluntarily shorten (vs lengthen) the homonymous muscle is associated with stronger anterior deltoid LLRs, and this enhancement of LLR gains is even stronger following a long preparatory delay (Fig. 8*B*, blue and purple bars in left panel vs right panel). Interestingly, like the case for SLRs of this muscle, there is no significant impact of target distance on the LLRs of the anterior deltoid (*p* = 0.064). However, there is an interaction effect between preparatory delay and target distance (*F*_(1,13)_ = 7.4, *p* = 0.018, and η^2^_p_ = 0.36), with *post hoc* analysis showing that all relevant comparisons were significantly different (all *p* < 0.003), with the exception of no significant impact of target distance when the delay was short (*p* = 0.099). There is also a weak but significant main effect of load (*F*_(2,26)_ = 3.7, *p* < 0.04, and η^2^_p_ = 0.22), an interaction effect between load and target distance (*F*_(2,26)_ = 4.7, *p* < 0.018, and η^2^_p_ = 0.27), and a further interaction effect among load, target distance, and direction distance (*F*_(2,26)_ = 4.9, *p* = 0.017, and η^2^_p_ = 0.27). The Tukey’s HSD test indicated a significant difference (*p* = 0.00014) as a function of target distance only when preparing muscle shortening in the no-load condition (Fig. 8*B*, blue vs purple targets in the no-load condition).

Equivalent results were obtained with regard to the posterior deltoid (Fig. 8*B*). The ANOVA indicated a significant main effect of target direction on posterior deltoid LLR (*F*_(1,13)_ = 78, *p* < 10^−5^, and η^2^_p_ = 0.86), again demonstrating that the preparation to move in one direction or another sets up different feedback gains. There is an interaction effect between target distance and direction (*F*_(1,13)_ = 8.7, *p* = 0.012, and η^2^_p_ = 0.4). However, Tukey’s HSD only indicated a significant impact of target direction (i.e., relative upregulation of LLR gains regardless whether the “shortening” target was near or far; both *p* < 0.0003). There is a weaker but significant main effect of load on posterior deltoid LLRs (*F*_(2,26)_ = 4.4, *p* = 0.023, and η^2^_p_ = 0.25) and an interaction effect between load and preparatory delay (*F*_(2,26)_ = 5.9, *p* = 0.008, and η^2^_p_ = 0.21). The Tukey’s HSD test indicated that a significant difference in LLR as a function of delay (i.e., higher LLR with longer delay) materialized only when the posterior deltoid was unloaded (*p* = 0.034). There is also an interaction effect among load, preparatory delay, and target direction (*F*_(2,26)_ = 3.9, *p* = 0.032, and η^2^_p_ = 0.23), with *post hoc* analyses indicating that the aforementioned impact of preparatory delay manifested only when preparing to shorten the unloaded posterior deltoid (*p* = 0.0036). Overall, therefore, there is a consistent pattern of LLR goal-directed tuning: relatively weaker/stronger reflex gains when preparing to reach targets requiring stretch/shortening of the homonymous muscle, with an amplification of this effect in the unloaded muscle if a sufficiently long preparatory delay is allowed.

## Discussion

The aim of our study was to investigate the goal-directed tuning of stretch reflex gains in the context of delayed reaching, and to further examine how such tuning depends on external loads and preparation duration. Participants were presented with four different targets (two directions and two distances) under three different background loads. At two delays (short and long) after the target was presented to the participants, a rapid position-dependent perturbation was applied to the arm to test whether stretch reflex gains were changed in preparation for the upcoming reaching movement. Despite identical background loads and perturbations to the dominant upper limb, both the SLR and LLR showed strong goal-directed modulation. Specifically, while target distance produced only small variations in the LLR gains, the direction of the prepared movement produced profound scaling of these responses. Moreover, as the delay between the target presentation and the perturbation increased, the LLRs showed stronger changes according to the target direction, especially when the muscles were unloaded (i.e., when an external force was first applied in the direction of homonymous muscle action). This long delay condition also revealed a consistent strong goal-directed tuning of SLR gains, particularly in the unloaded condition. As with the LLR, we demonstrate that the SLR shows a clear modulation according to the presented target direction in all three analyzed muscles, increasing in gain when the muscles will be shortened by the upcoming movement, and decreasing when they are expected to be lengthened. Overall, we show that preparing to reach a target produces a congruent modulation of the stretch reflex responses at both the SLR and LLR epochs, suggesting the independent involvement of the fusimotor system in movement preparation. A long enough preparation (>250 ms) and muscle unloading (assistive loading) appear to trigger or enhance this independent preparatory control of reflex muscle stiffness (Figs. 6, 7).

### SLRs

With the exception of long-term reward-based training (Wolpaw, 1982), most previous research examining the modulation of SLRs has shown little task-relevant modulation of feedback gains. In particular, until recently, SLRs were considered to mainly exhibit gain scaling or presynaptic modulation according to tasks. Gain scaling is the modulation of the feedback responses with the background activity or load (Matthews, 1986; Pruszynski et al., 2009). In addition to gain scaling, several articles have provided evidence using H-reflexes that presynaptic inhibition on the Ia afferents can affect the SLR (Capaday and Stein, 1987; Nielsen and Kagamihara, 1993; Stein, 1995; Perez et al., 2005). In general, these studies have shown modulation of the H-reflex for different tasks such as standing or walking, or during cocontraction, independent of the specific level of muscle activity. The modulation of the H-reflex during such tasks suggested a descending modulation of the SLR from presynaptic inhibition. More recently, Weiler et al. (2019, 2021) have demonstrated that wrist posture influences the SLRs at the elbow in a task-relevant manner, suggesting that spinal circuits are capable of integrating information from nearby joints. In a similar manner, although gain scaling is normally described within a single muscle or motor neuron pool, a recent study has shown SLR modulation that depends also on synergistic muscle activity across the shoulder joint (Nicolozakes et al., 2022). This modulation of SLR in the absence of movement or movement planning is compatible with the antagonistic muscle balance hypothesis (Dimitriou, 2014), which has been supported independently (Villamar et al., 2023). Finally, it has also been shown recently that, despite the same initial posture, there is a goal-directed modulation of SLR gains when preparing to reach with the dominant limb (Papaioannou and Dimitriou, 2021). Specifically, when the perturbation stretching the homonymous muscle occurs in the direction of the cued target, there is a smaller SLR than if the same perturbation acts in the direction opposite to the target. Here we confirm this initial finding, showing again that the SLR is consistently modulated according to target direction.

In the absence of equivalent differences in preperturbation muscle activity, the effect of target direction on SLR suggests that movement preparation affects muscle spindle tuning, changing the SLR feedback gain during preparation via the independent control of fusimotor neurons. Indeed, SLR modulation across muscles relied on there being sufficient time between the target presentation and the perturbation, longer than the minimum time required for shaping reflex responses via selective CNS processing of sensory signals (Scott, 2016). In turn, this demonstrates that the preparatory modulation of reflex muscle stiffness requires sufficient time to completely unfold (>250 ms), which fits with the known slow-evolving nature of net fusimotor impact on spindle afferent responses (Crowe and Matthews, 1964a, b). The proposal that independent fusimotor control is involved in movement preparation is also compatible with the demonstrated preparatory changes in the somatosensory cortex (Ariani et al., 2022) and the notion that feedback controllers are loaded before movement onset (Ahmadi-Pajouh et al., 2012).

While we suggest that the goal-directed modulation of the SLR in our movement preparation task occurs through independent fusimotor control, there is still a possibility that the observed changes occurred through presynaptic inhibition. While this cannot be ruled out with the current study, we believe that this is unlikely for several reasons. First, presynaptic inhibition would be expected to provide consistent modulation of the SLR regardless of the background activity of the muscle, whereas in our study this did not occur when the muscle was directly loaded. Second, there is already evidence for a goal-directed preparatory modulation of muscle spindles in delayed reach (Papaioannou and Dimitriou, 2021), and more importantly this modulation matches the temporal evolution of the observed SLR tuning, where stronger modulation is found for delays >250 ms. We therefore propose that the goal-directed tuning of SLR gain arises primarily through changes in γ drive. The classic view that muscle spindle sensitivity relates to α–γ coactivation (Vallbo, 1970; Vallbo et al., 1979) is changing to a more dynamic view where the fusimotor system allows flexible signal processing at the periphery (Dimitriou, 2022). We find stronger goal-directed modulation of SLRs when the muscle is unloaded. We hypothesize that this arises because antagonist loading is accompanied by top-down reciprocal inhibition of lower motor neurons of the muscle, including γ motor neurons (Dimitriou, 2014). The stronger goal-directed effects then arise as the independent goal-directed control of dynamic γ motor neurons occurs on top of this blanket reciprocal inhibition of lower motor neurons that accompanies muscle unloading.

### LLRs

It is well known that LLRs can vary according to task goals (Akazawa et al., 1983; Kimura et al., 2006; Pruszynski et al., 2008; Nashed et al., 2012). Early work demonstrated that simple commands to the participants such as to “relax” or “resist” the perturbation produced large variations in reflex responses (Crago et al., 1976; Rothwell et al., 1980). This was further refined by placing different targets either in the direction of the perturbation or in the opposite direction to see whether the stretch reflex responses would be modified by the location and distance of the target (Pruszynski et al., 2008). The long-latency responses were shown to modulate strongly according to the target location, increasing when the perturbation was in the opposite direction to the target and decreasing when perturbed in the direction of the target. Accordingly, it was recently shown that LLR gains are modulated when preparing to reach toward a cued target (Papaioannou and Dimitriou, 2021). In the present study, we examined how these responses are affected by movement distance. Here, we found that the LLRs showed strong modulation according to the direction of the planned future movement. That is, if the perturbation was in the direction opposite to the target of the upcoming movement, the LLR gain was increased, but if the perturbation was in the direction of the target, it was decreased (Fig. 7). When the delay between the target presentation and the perturbation was relatively short (250 ms), there was little effect of target distance or background load on the responses. However, additional preparation time (i.e., the long delay) was associated with stronger goal-directed modulation of LLRs according to both target distance (especially for perturbations opposite to the target direction) and background load.

### Effect of preparatory delay

In our study, there are strong differences according to the preparatory delay between target presentation and perturbation, both in the SLR and LLR epochs. Goal-directed differences at the SLR epoch were consistently observed only following the relatively long preparatory delay (Fig. 6). In the LLR epoch, for short preparatory delays we find clear tuning of the reflex gains according to the target direction, but there is little to no effect of target distance or background load. In contrast, for longer delays we find tuning of the feedback gains according to the target distance and modulation according to the background load, and an even stronger impact of target direction (Fig. 7). This goal-directed modulation of feedback gains is appropriately tuned for the differences in the goals. That is, targets that are further away require stronger feedback gains. The difference in responses for short-delay and long-delay conditions suggests that sufficient time is needed to determine how the feedback gains should modulate for further targets and different environmental dynamics. However, the current study only examined the stretch reflex modulation for two different delays (250 and 750 ms); we cannot define the minimum time required for the full expression of goal-directed tuning of stretch reflexes. Further studies are needed to determine in more detail how such reflex modulation evolves over preparatory time.

Overall, we find larger differences in the goal-directed feedback gains for longer preparatory delays. However, even more interesting is the difference between the LLR and SLR tuning as a function of preparatory delay. Our results show that even the relatively short delay of 250 ms was sufficient to systematically evoke tuning of the LLRs according to target direction. However, no consistent modulation of the SLR was found for target direction or distance at this short delay. Instead, only for the longer delay did we find evidence of SLR modulation as a function of target direction. The above, and the presence of a stronger effect of target direction on LLR gains following a long delay, support the proposal that a slower-evolving mechanism (i.e., the independent fusimotor control of spindles) is also involved in the preparatory modulation of stretch reflex gains. Indeed, it has been shown previously that muscle spindle Ia signals (and hence their fusimotor control) can affect LLRs (Hunter et al., 1988; Fellows et al., 1993) that are likely mediated by both spinal and supraspinal circuits (Cheney and Fetz, 1984; Pruszynski et al., 2011b; Kurtzer, 2014; Soteropoulos and Baker, 2020).

### Effect of background loading

Many previous studies of stretch reflex modulation preloaded the muscles before applying perturbations. That is, they provide a background load to excite the muscle that they will stretch to elicit strong EMG responses. However, it can be seen in our study that such preloading to increase muscle activity also strongly affects the degree of reflex modulation. Specifically, there was no goal-directed modulation of SLR observed when the muscle was heavily loaded. This is likely because of the automatic gain-scaling feature, which tends to saturate the SLR (Matthews, 1986; Pruszynski et al., 2009). However, in the no-load or unloaded conditions we find goal-directed modulation of the SLRs. These conditions reflect the everyday case of reaching to grasp an object, where the muscles are unloaded before the movement initiation. In contrast, preloading a muscle increases the α motor neuron drive, exciting the motoneuron pools, and appears to limit our ability to see such goal-directed modulation at the SLR epoch. We suggest that it is critical for future studies to be performed under a range of loaded and unloaded conditions to examine the true range of feedback modulation. Moreover, the effects of background loading are even apparent within the LLR interval. In particular, we found the strongest goal-directed modulation of LLR and SLRs to occur when the muscle was unloaded i.e., in cases where a background—“assistive”—load was applied in the direction of homonymous muscle action. That goal-directed tuning of reflex muscle stiffness is enhanced by muscle unloading may partly account for the favorable impact of assistive loading for the purposes of motor rehabilitation (Wu et al., 2014).

### Effect of target parameters

In the current study, we examine the goal-oriented modulation of stretch reflex responses by examining the effect of target distance. There is a long-standing history of examining changes in muscle activation with target distance (Buneo et al., 1994; Kaminski et al., 1995; Tyler and Karst, 2004), with timing and intensity varying as the distance increases. Scaling is also found for visuomotor responses, with variations in feedback gains to visual perturbations of the cursor with distance (Dimitriou et al., 2013) that can be explained by the time required to reach the target (Česonis and Franklin, 2020, 2022). However, there is very limited information regarding how target distance influences stretch reflex responses. In one study (Pruszynski et al., 2008), target distance was used to control the degree of resistance to the perturbations, showing a strong increase in LLR stretch gains with target distance for elbow movements.

Here we show similar goal-directed modulation of the LLR reflexes, particularly when the muscles are stretched against the direction of the target. However, we find that there is little or no variation with target distance when the homonymous muscle is lengthened in the direction of the cued target. Interestingly, we also find evidence for some target distance modulation of the SLR reflex when the muscles are stretched after longer preparatory periods. Nevertheless, the demonstrated impact of target direction on SLR gains strongly suggests that goal-directed tuning of spindles is a basic component of reach preparation (Papaioannou and Dimitriou, 2021). That is, reach preparation includes implementation of a plan at the periphery (i.e., modification of spindle gains) and does not only involve motor planning or otherwise priming of the CNS. In terms of the effect of target direction on reflex gains (relative downregulation if the reach involves stretch of the homonymous muscle), it was more universal in our experimental results and is shown in all three muscles. This result was rather easy to interpret as facilitating goal-directed behavior, showing the compliance along the desired movement direction. However, at the same time we also found quite a robust effect of target distance on the pectoralis major SLR, which showed higher gains for far targets, but this effect was not consistent across muscles.

### Reflex modulation and stiffness

The stiffness or compliance of the body has long been acknowledged as an important factor in motor control (Hogan, 1984; McIntyre et al., 1995; Wolpert and Flanagan, 2010; Franklin and Wolpert, 2011), but most reaching studies examine changes because of cocontraction (Osu et al., 2002; Franklin et al., 2007; Franklin and Franklin, 2021) or changing posture (Trumbower et al., 2009; Lametti and Ostry, 2010; Franklin et al., 2013). However, reflexes also contribute significantly to the stiffness of the muscles and limbs (Crago et al., 1976; Nichols and Houk, 1976; Hoffer and Andreassen, 1981; Akazawa et al., 1983; Kearney et al., 1997). While stiffness because of cocontraction or posture provides an instantaneous response to a perturbation, reflexive contributions to stiffness act at a delay because of neural transmission delays and electromechanical delays of muscles (Ito et al., 2004). Because of this additional delay, reflex modulation is often thought to contribute more to the stiffness and stability in body posture tasks (Loram et al., 2011) than in object manipulation (Morasso, 2011), although both contribute (Franklin et al., 2007). Here we show evidence that there can be independent control of stretch reflexes via the fusimotor system, meaning that there can be task-dependent control of the rapid SLR for delayed reaching. These responses can modulate the force responses to perturbations quickly, as they do not have to wait for sensory input to reach the brain, be selectively processed in the brain, and then be passed back to the muscles. Indeed, independent fusimotor control provides true online goal-directed stiffness control, and with much lower energy consumption than cocontraction. As exemplified by the effect of preparatory delay in our study, such modulation does require some time to prepare, due at least in part to the slower nature of the γ fusimotor system.

### Summary

In the current study, we sought to examine the preparatory modulation of short-latency and long-latency stretch reflex responses in the dominant upper limb. While target distance was associated with relatively small variations in reflex gains, both short-latency and long-latency gains were strongly modulated as a function of target direction, in a manner that facilitated the upcoming voluntary movement. This goal-directed tuning of reflex gains was triggered or enhanced when the preparatory delay was sufficiently long (>250 ms) and the homonymous muscle was unloaded (i.e., when a background load was first applied in the direction of homonymous muscle action; assistive loading). The results support the proposal that reach preparation also involves the goal-directed modulation of reflexive stiffness, likely via the independent control of fusimotor neurons.

## Synthesis

Reviewing Editor: Mark Laubach, American University Decisions are customarily a result of the Reviewing Editor and the peer reviewers coming together and discussing their recommendations until a consensus is reached. When revisions are invited, a fact-based synthesis statement explaining their decision and outlining what is needed to prepare a revision will be listed below. The following reviewer(s) agreed to reveal their identity: Eric Perreault.

Your manuscript was reviewed by two experts and a number of issues were raised that need to be addressed before a decision can be made about your paper. Please revise the manuscript to address all points in the reviews below. Thank you for sending your work to eNeuro.

Reviewer 1

This study evaluated how movement preparation influences short and long latency reflexes elicited in the dominant upper limb. Measurements were made at different levels of background load and for planned movements that differed in direction and extent. The results demonstrate a clear impact of intended movement direction on the short and long latency (SLR and LLR) reflexes, particularly when the stretched muscle was inactive prior to stretch. The results are novel and interesting, particularly those for the SLR as these are amongst the first results demonstrating a clear preparatory effect on the SLR. I have only a few suggestions that I think would clarify the results and strengthen the impact of this manuscript.

Major Comments

-----------------

The results demonstrating modulation of the SLR based on intended movement direction are very nice. The authors propose that the differences result from activation of the fusimotor system. If so, I would expect to see modulation at the onset of SLR activity. This clearly occurs in the results for a single participant (Fig. 3) but not in the group results (Figs. 4 & 5). The difference may come from averaging EMGs from all subjects. The authors are strongly encouraged to estimate the onset of SLR reflex modulation for each subject to determine if those who exhibit strong modulation do so for the entire period of the SLR.

The use of a z-transform to normalize rectified EMG signals is an odd choice given that the z-transform is commonly used for normally distributed data, but rectified signals will have a very different distribution. The main impact could be a difficulty detecting changes in mean values since the transform will likely use different mean shifts for each subject. The authors should verify that the pre-perturbation results are unchanged by a more traditional normalization such as MVC-normalization. As the authors note, rigorously testing for differences in the pre-perturbation EMG is essential before the differences in reflex responses can be interpreted.

The fact that SLR modulation is only observed consistently during unloaded conditions is interesting and important, as it positions this work in the context of the many previous studies that did not observe SLR modulation under similar conditions. The loaded condition most relevant to previous work is currently not included (Fig. 6). Adding this final condition would increase the utility of this manuscript, even of the results are null.

A rationale for studying reflexes with a well-controlled level of non-zero background activity has been to reduce experimental variability by controlling motoneuron pool excitability. Was there any evidence that the reflexes on the loaded condition were less variable than those in the other two conditions?

The authors focus on a single explanation for the observed results, which is task-dependent changes in fusimotor drive. Given that the contributions from fusimotor drive were not directly assessed in this study, a more thorough discussion of alternative interpretations should be provided including task-dependent changes in presynaptic inhibition (e.g. Stein, 1995).

Minor Comments

-----------------

The last paragraph of the Introduction would be strengthened by stating a hypothesis to be tested or at least a clear objective prior to summarizing the results.

At least the most important statistical comparisons should provide mean and confidence intervals of the quantity of interest not just the p-value, which can be highly misleading (Nuzzo, 2014).

The error bars in Fig. 6 do not seem to match the results in the text. Presumably this is because the 95% confidence intervals in the figure are for the mean in each group rather than the mean of the subject-specific comparisons reported in the text. Some clarification for the reader would be helpful.

Line 57. “planed” should be “planned”

Line 77. “investigating” should be “investigated”

Lines 470-474. Recent work by Nicolozakes (2022) showing task-dependent modulation of SLRs at the shoulder should be cited as evidence for SLR modulation even in the absence of a planned movement.

References

-----------

Nicolozakes, C. P., Sohn, M. H., Baillargeon, E. M., Lipps, D. B., & Perreault, E. J. (2022). Stretch reflex gain scaling at the shoulder varies with synergistic muscle activity. J Neurophysiol, 128(5), 1244-1257. https://doi.org/10.1152/jn.00259.2022

Nuzzo, R. (2014). Statistical errors: P values, the “gold standard” of statistical validity, are not as reliable as many scientists assume. Nature, 506(7487), 150-152. https://doi.org/10.1136/bmj.1.6053.66

Stein, R. B. (1995). Presynaptic inhibition in humans. Prog Neurobiol, 47(6), 533-544. https://doi.org/10.1016/0301-0082(95)00036-4

Reviewer 2

his study has examined how the stretch reflex gain was modulated depending on the upcoming reaching movement. The authors demonstrated that SLR and LLR were modulated with the target direction. This reflex tuning with the target direction was more pronounced when the preparatory delay after the target presentation became longer. Furthermore, they found that the target distance influenced the SLR for the pectoralis muscle for the unloaded condition. The authors concluded that the fusimotor neurons are controlled independently from the (alpha) motoneurons.

Major points

1. Using the same experimental procedure, the authors (possibly) have already demonstrated the effect of target and load directions and the preparatory delay on the SLR and LLR (Sci Adv 2021). Thus, new aspects in this study were limited to the effect of target distance. Considering that the effect of target distance was relatively weak, the novelty of this work was also limited. I still thought the results were valuable enough to be reported, but the authors should describe explicitly that this was an extended study from their previous work (I can understand that the authors could not say so in the reviewing process because of the double-blind review procedure in eNeuro,).

2. The authors described that the task-dependent modulation of SLR has been demonstrated only in a few studies (Weiler et al. Nat Neurosci 2019, Papaioannou & Dimitriou 2021). In addition, the authors considered that the independent control of the fusimotor neurons derived the modulation. However, the task-dependent modulation of the mono-synaptic reflex was well documented in the previous studies (e.g., Capaday & Stein, Nilsen et al., Perez et al.). They also reported that a presynaptic inhibition on the Ia afferent nerves was involved in this modulation, suggesting that the control of the fusimotor neuron is not the only factor to modulate the monosynaptic reflex. The related studies should be mentioned and discussed.

3. The load condition was excluded from the analysis of SLR. This procedure looks strange. The authors could include this condition in the ANOVA?

4. The instruction for the reaching task needed to be clarified. The participants started reaching movements only after the perturbation was imposed? If so, the participants did not have to prepare for the upcoming movement sufficiently, which might obscure the effect of the target distance?

5. Figure 4 and 5: the colors in the legend for the ‘far’ target (A, D) seemed opposite (yellow vs. blue).

6. Line 324: ‘.’ is necessary.

## References

[B1] Ahmadi-Pajouh MA, Towhidkhah F, Shadmehr R (2012) Preparing to reach: selecting an adaptive long-latency feedback controller. J Neurosci 32:9537–9545. 10.1523/JNEUROSCI.4275-11.2012 22787039 PMC3407880

[B2] Akazawa K, Milner TE, Stein RB (1983) Modulation of reflex EMG and stiffness in response to stretch of human finger muscle. J Neurophysiol 49:16–27. 10.1152/jn.1983.49.1.16 6827293

[B3] Ariani G, Pruszynski JA, Diedrichsen J (2022) Motor planning brings human primary somatosensory cortex into action-specific preparatory states. Elife 11:e69517. 10.7554/eLife.69517 35018886 PMC8786310

[B4] Batista AP, Santhanam G, Yu BM, Ryu SI, Afshar A, Shenoy KV (2007) Reference frames for reach planning in macaque dorsal premotor cortex. J Neurophysiol 98:966–983. 10.1152/jn.00421.2006 17581846

[B5] Buneo CA, Soechting JF, Flanders M (1994) Muscle activation patterns for reaching: the representation of distance and time. J Neurophysiol 71:1546–1558. 10.1152/jn.1994.71.4.1546 8035234

[B6] Brooks VB (1979) Motor programs revisited. In: Posture and movement (Talbot RE, Humphrey DR, eds), pp 13–49. New York: Raven.

[B7] Capaday C, Stein RB (1987) Difference in the amplitude of the human soleus H reflex during walking and running. J Physiol 392:513–522. 10.1113/jphysiol.1987.sp016794 3446790 PMC1192318

[B8] Česonis J, Franklin DW (2020) Time-to-target simplifies optimal control of visuomotor feedback responses. eNeuro 7:ENEURO.0514-19.2020. 10.1523/ENEURO.0514-19.2020 PMC7189480 32213555

[B9] Česonis J, Franklin DW (2022) Contextual cues are not unique for motor learning: task-dependant switching of feedback controllers. PLoS Comput Biol 18:e1010192. 10.1371/journal.pcbi.1010192 35679316 PMC9217135

[B10] Cheney PD, Fetz EE (1984) Corticomotoneuronal cells contribute to long-latency stretch reflexes in the rhesus monkey. J Physiol 349:249–272. 10.1113/jphysiol.1984.sp015155 6737294 PMC1199336

[B11] Churchland MM, Santhanam G, Shenoy KV (2006) Preparatory activity in premotor and motor cortex reflects the speed of the upcoming reach. J Neurophysiol 96:3130–3146. 10.1152/jn.00307.2006 16855111

[B12] Churchland MM, Cunningham JP, Kaufman MT, Ryu SI, Shenoy KV (2010) Cortical preparatory activity: representation of movement or first cog in a dynamical machine? Neuron 68:387–400. 10.1016/j.neuron.2010.09.015 21040842 PMC2991102

[B13] Churchland MM, Cunningham JP, Kaufman MT, Foster JD, Nuyujukian P, Ryu SI, Shenoy KV (2012) Neural population dynamics during reaching. Nature 487:51–56. 10.1038/nature11129 22722855 PMC3393826

[B14] Cluff T, Scott SH (2015) Apparent and actual trajectory control depend on the behavioral context in upper limb motor tasks. J Neurosci 35:12465–12476. 10.1523/JNEUROSCI.0902-15.2015 26354914 PMC6605398

[B15] Corneil BD, Olivier E, Munoz DP (2004) Visual responses on neck muscles reveal selective gating that prevents express saccades. Neuron 42:831–841. 10.1016/s0896-6273(04)00267-3 15182721

[B16] Crago PE, Houk JC, Hasan Z (1976) Regulatory actions of human stretch reflex. J Neurophysiol 39:925–935. 10.1152/jn.1976.39.5.925 978238

[B17] Crowe A, Matthews PB (1964a) The effects of stimulation of static and dynamic fusimotor fibres on the response to stretching of the primary endings of muscle spindles. J Physiol 174:109–131. 10.1113/jphysiol.1964.sp007476 14228607 PMC1368929

[B18] Crowe A, Matthews PB (1964b) Further studies of static and dynamic fusimotor fibres. J Physiol 174:132–151. 10.1113/jphysiol.1964.sp007477 14228608 PMC1368930

[B19] Dimitriou M (2014) Human muscle spindle sensitivity reflects the balance of activity between antagonistic muscles. J Neurosci 34:13644–13655. 10.1523/JNEUROSCI.2611-14.2014 25297092 PMC6608384

[B20] Dimitriou M (2016) Enhanced muscle afferent signals during motor learning in humans. Curr Biol 26:1062–1068. 10.1016/j.cub.2016.02.030 27040776

[B21] Dimitriou M (2018) Task-dependent modulation of spinal and transcortical stretch reflexes linked to motor learning rate. Behav Neurosci 132:194–209. 10.1037/bne0000241 29809047

[B22] Dimitriou M (2021) Crosstalk proposal: there is much to gain from the independent control of human muscle spindles. J Physiol 599:2501–2504. 10.1113/JP281338 33749831

[B23] Dimitriou M (2022) Human muscle spindles are wired to function as controllable signal-processing devices. eLife 11:e78091. 10.7554/eLife.78091 35829705 PMC9278952

[B24] Dimitriou M, Franklin DW, Wolpert DM (2012) Task-dependent coordination of rapid bimanual motor responses. J Neurophysiol 107:890–901. 10.1152/jn.00787.2011 22072514 PMC3289469

[B25] Dimitriou M, Wolpert DM, Franklin DW (2013) The temporal evolution of feedback gains rapidly update to task demands. J Neurosci 33:10898–10909. 10.1523/JNEUROSCI.5669-12.2013 23804109 PMC3724995

[B26] Fellows SJ, Dömges F, Töpper R, Thilmann AF, Noth J (1993) Changes in the short- and long-latency stretch reflex components of the triceps surae muscle during ischaemia in man. J Physiol 472:737–748. 10.1113/jphysiol.1993.sp019970 8145169 PMC1160510

[B27] Franklin S, Franklin DW (2021) Feedback gains modulate with motor memory uncertainty. arXiv:2008.07574. 10.48550/arXiv.2008.07574.

[B28] Franklin DW, Wolpert DM (2011) Computational mechanisms of sensorimotor control. Neuron 72:425–442. 10.1016/j.neuron.2011.10.006 22078503

[B29] Franklin DW, Liaw G, Milner TE, Osu R, Burdet E, Kawato M (2007) Endpoint stiffness of the arm is directionally tuned to instability in the environment. J Neurosci 27:7705–7716. 10.1523/JNEUROSCI.0968-07.2007 17634365 PMC6672883

[B30] Franklin DW, Selen LP, Franklin S, Wolpert DM (2013) Selection and control of limb posture for stability. In: 2013 35th Annual International Conference of the IEEE Engineering in Medicine and Biology Society (EMBC), pp 5626–5629. Piscataway, NJ: IEEE. 10.1109/EMBC.2013.6610826 PMC6103437 24111013

[B31] Ghez C, Favilla M, Ghilardi MF, Gordon J, Bermejo R, Pullman S (1997) Discrete and continuous planning of hand movements and isometric force trajectories. Exp Brain Res 115:217–233. 10.1007/pl00005692 9224851

[B32] Green DM, Swets JA (1966) Signal detection theory and psychophysics. New York: Wiley.

[B33] Hammond PH (1956) The influence of prior instruction to the subject on an apparently involuntary neuro-muscular response. J Physiol 132:17–18. 13320395

[B34] Hocherman S, Wise SP (1991) Effects of hand movement path on motor cortical activity in awake, behaving rhesus monkeys. Exp Brain Res 83:285–302. 10.1007/BF00231153 2022240

[B35] Hoffer JA, Andreassen S (1981) Regulation of soleus muscle stiffness in premammillary cats: intrinsic and reflex components. J Neurophysiol 45:267–285. 10.1152/jn.1981.45.2.267 6780665

[B36] Hogan N (1984) An organizing principle for a class of voluntary movements. J Neurosci 4:2745–2754. 10.1523/JNEUROSCI.04-11-02745.1984 6502203 PMC6564718

[B37] Hunter JP, Ashby P, Lang AE (1988) Afferents contributing to the exaggerated long latency reflex response to electrical stimulation in Parkinson's disease. J Neurol Neurosurg Psychiatry 51:1405–1410. 10.1136/jnnp.51.11.1405 2853207 PMC1032811

[B38] Ito T, Murano EZ, Gomi H (2004) Fast force-generation dynamics of human articulatory muscles. J Appl Physiol (1985) 96:2318–2324. 10.1152/japplphysiol.01048.2003 14990556

[B39] Kaminski TR, Bock C, Gentile AM (1995) The coordination between trunk and arm motion during pointing movements. Exp Brain Res 106:457–466. 10.1007/BF00231068 8983989

[B40] Kearney RE, Stein RB, Parameswaran L (1997) Identification of intrinsic and reflex contributions to human ankle stiffness dynamics. IEEE Trans Biomed Eng 44:493–504. 10.1109/10.581944 9151483

[B41] Kimura T, Haggard P, Gomi H (2006) Transcranial magnetic stimulation over sensorimotor cortex disrupts anticipatory reflex gain modulation for skilled action. J Neurosci 26:9272–9281. 10.1523/JNEUROSCI.3886-05.2006 16957083 PMC6674505

[B42] Kurtzer IL (2014) Long-latency reflexes account for limb biomechanics through several supraspinal pathways. Front Integr Neurosci 8:99. 10.3389/fnint.2014.00099 25688187 PMC4310276

[B43] Kutas M, Donchin E (1974) Studies of squeezing: handedness, responding hand, response force, and asymmetry of readiness potential. Science 186:545–548. 10.1126/science.186.4163.545 4469679

[B44] Lametti DR, Ostry DJ (2010) Postural constraints on movement variability. J Neurophysiol 104:1061–1067. 10.1152/jn.00306.2010 20554837 PMC2941207

[B45] Lee H, Perreault EJ (2019) Stabilizing stretch reflexes are modulated independently from the rapid release of perturbation-triggered motor plans. Sci Rep 9:13926. 10.1038/s41598-019-50460-1 31558754 PMC6763490

[B46] Loram ID, Gollee H, Lakie M, Gawthrop PJ (2011) Human control of an inverted pendulum: is continuous control necessary? Is intermittent control effective? Is intermittent control physiological? J Physiol 589:307–324. 10.1113/jphysiol.2010.194712 21098004 PMC3043535

[B47] Marsden CD, Merton PA, Morton HB (1972) Servo action in human voluntary movement. Nature 238:140–143. 10.1038/238140a0 4558452

[B48] Matthews PB (1986) Observations on the automatic compensation of reflex gain on varying the pre-existing level of motor discharge in man. J Physiol 374:73–90. 10.1113/jphysiol.1986.sp016066 3746703 PMC1182707

[B49] McIntyre J, Gurfinkel EV, Lipshits MI, Droulez J, Gurfinkel VS (1995) Measurements of human force control during a constrained arm motion using a force-actuated joystick. J Neurophysiol 73:1201–1222. 10.1152/jn.1995.73.3.1201 7608766

[B50] Messier J, Kalaska JF (2000) Covariation of primate dorsal premotor cell activity with direction and amplitude during a memorized-delay reaching task. J Neurophysiol 84:152–165. 10.1152/jn.2000.84.1.152 10899193

[B51] Morasso P (2011) “Brute force” vs. “gentle taps” in the control of unstable loads. J Physiol 589:459–460. 10.1113/jphysiol.2010.203604 21285028 PMC3055535

[B52] Nashed JY, Crevecoeur F, Scott SH (2012) Influence of the behavioral goal and environmental obstacles on rapid feedback responses. J Neurophysiol 108:999–1009. 10.1152/jn.01089.2011 22623483

[B53] Nashed JY, Crevecoeur F, Scott SH (2014) Rapid online selection between multiple motor plans. J Neurosci 34:1769–1780. 10.1523/JNEUROSCI.3063-13.2014 24478359 PMC8186509

[B54] Nichols TR, Houk JC (1976) Improvement in linearity and regulation of stiffness that results from actions of stretch reflex. J Neurophysiol 39:119–142. 10.1152/jn.1976.39.1.119 1249597

[B55] Nicolozakes CP, Sohn MH, Baillargeon EM, Lipps DB, Perreault EJ (2022) Stretch reflex gain scaling at the shoulder varies with synergistic muscle activity. J Neurophysiol 128:1244–1257. 10.1152/jn.00259.2022 36224165 PMC9662809

[B56] Nielsen J, Kagamihara Y (1993) The regulation of presynaptic inhibition during co-contraction of antagonistic muscles in man. J Physiol 464:575–593. 10.1113/jphysiol.1993.sp019652 8229819 PMC1175403

[B57] Osu R, Franklin DW, Kato H, Gomi H, Domen K, et al. (2002) Short- and long-term changes in joint co-contraction associated with motor learning as revealed from surface EMG. J Neurophysiol 88:991–1004. 10.1152/jn.2002.88.2.991 12163548

[B58] Papaioannou S, Dimitriou M (2021) Goal-dependent tuning of muscle spindle receptors during movement preparation. Sci Adv 7:eabe0401. 33627426 10.1126/sciadv.abe0401 PMC7904268

[B59] Perez MA, Lungholt BK, Nielsen JB (2005) Presynaptic control of group Ia afferents in relation to acquisition of a visuo-motor skill in healthy humans. J Physiol 568:343–354. 10.1113/jphysiol.2005.089904 16051628 PMC1474778

[B60] Pruszynski JA, Kurtzer I, Scott SH (2008) Rapid motor responses are appropriately tuned to the metrics of a visuospatial task. J Neurophysiol 100:224–238. 10.1152/jn.90262.2008 18463184

[B61] Pruszynski JA, Kurtzer I, Lillicrap TP, Scott SH (2009) Temporal evolution of “automatic gain-scaling”. J Neurophysiol 102:992–1003. 10.1152/jn.00085.2009 19439680 PMC2724331

[B62] Pruszynski JA, Kurtzer I, Scott SH (2011a) The long-latency reflex is composed of at least two functionally independent processes. J Neurophysiol 106:449–459. 10.1152/jn.01052.2010 21543751

[B63] Pruszynski JA, Kurtzer I, Nashed JY, Omrani M, Brouwer B, Scott SH (2011b) Primary motor cortex underlies multi-joint integration for fast feedback control. Nature 478:387–390. 10.1038/nature10436 21964335 PMC4974074

[B64] Rothwell JC, Traub MM, Marsden CD (1980) Influence of voluntary intent on the human long-latency stretch reflex. Nature 286:496–498. 10.1038/286496a0 7402329

[B65] Scott SH (2016) A functional taxonomy of bottom-up sensory feedback processing for motor actions. Trends Neurosci 39:512–526. 10.1016/j.tins.2016.06.001 27378546

[B66] Shadmehr R, Krakauer JW (2008) A computational neuroanatomy for motor control. Exp Brain Res 185:359–381. 10.1007/s00221-008-1280-5 18251019 PMC2553854

[B67] Shemmell J, Krutky MA, Perreault EJ (2010) Stretch sensitive reflexes as an adaptive mechanism for maintaining limb stability. Clin Neurophysiol 121:1680–1689. 10.1016/j.clinph.2010.02.166 20434396 PMC2932821

[B68] Soteropoulos DS, Baker SN (2020) Long-latency responses to a mechanical perturbation of the index finger have a spinal component. J Neurosci 40:3933–3948. 10.1523/JNEUROSCI.1901-19.2020 32245828 PMC7219296

[B69] Stein RB (1995) Presynaptic inhibition in humans. Prog Neurobiol 47:533–544. 10.1016/0301-0082(95)00036-4 8787034

[B70] Sternberg S, Monsell S, Knoll RL, Wright CE (1978) The latency and duration of rapid movement sequences: comparisons of speech and typewriting. In: Information processing in motor control and learning (Stelmach GE, ed), pp 117–152. New York: Academic.

[B71] Tanji J, Evarts EV (1976) Anticipatory activity of motor cortex neurons in relation to direction of an intended movement. J Neurophysiol 39:1062–1068. 10.1152/jn.1976.39.5.1062 824409

[B72] Todorov E, Jordan MI (2002) Optimal feedback control as a theory of motor coordination. Nat Neurosci 5:1226–1235. 10.1038/nn963 12404008

[B73] Trumbower RD, Krutky MA, Yang BS, Perreault EJ (2009) Use of self-selected postures to regulate multi-joint stiffness during unconstrained tasks. PLoS One 4:e5411. 10.1371/journal.pone.0005411 19412540 PMC2671603

[B74] Tyler AE, Karst GM (2004) Timing of muscle activity during reaching while standing: systematic changes with target distance. Gait Posture 20:126–133. 10.1016/j.gaitpost.2003.07.001 15336281

[B75] Vallbo AB (1970) Discharge patterns in human muscle spindle afferents during isometric voluntary contractions. Acta Physiol Scand 80:552–566. 10.1111/j.1748-1716.1970.tb04823.x 4250202

[B76] Vallbo AB, Hagbarth KE, Torebjörk HE, Wallin BG (1979) Somatosensory, proprioceptive, and sympathetic activity in human peripheral nerves. Physiol Rev 59:919–957. 10.1152/physrev.1979.59.4.919 227005

[B77] Villamar Z, Ludvig D, Perreault EJ (2023) Short latency stretch reflexes depend on the balance of activity in agonist and antagonist muscles during ballistic elbow movements. J Neurophysiol 129:7–16. 10.1152/jn.00171.2022 36475940 PMC9799151

[B78] Wagner MJ, Smith MA (2008) Shared internal models for feedforward and feedback control. J Neurosci 28:10663–10673. 10.1523/JNEUROSCI.5479-07.2008 18923042 PMC6671341

[B79] Weiler J, Gribble PL, Pruszynski JA (2019) Spinal stretch reflexes support efficient hand control. Nat Neurosci 22:529–533. 10.1038/s41593-019-0336-0 30742115

[B80] Weiler J, Gribble PL, Pruszynski JA (2021) Spinal stretch reflexes support efficient control of reaching. J Neurophysiol 125:1339–1347. 10.1152/jn.00487.2020 33689494

[B81] Wise SP (1985) The primate premotor cortex: past, present, and preparatory. Annu Rev Neurosci 8:1–19. 10.1146/annurev.ne.08.030185.000245 3920943

[B82] Wolpaw JR (1982) Change in short-latency response to limb displacement in primates. Fed Proc 41:2156–2159. 7075789

[B83] Wolpert DM, Flanagan JR (2010) Motor learning. Curr Biol 20:R467–R472. 10.1016/j.cub.2010.04.035 20541489

[B84] Wu M, Landry JM, Kim J, Schmit BD, Yen SC, Macdonald J (2014) Robotic resistance/assistance training improves locomotor function in individuals poststroke: a randomized controlled study. Arch Phys Med Rehabil 95:799–806. 10.1016/j.apmr.2013.12.021 24440365 PMC4076161

[B85] Yang L, Michaels JA, Pruszynski JA, Scott SH (2011) Rapid motor responses quickly integrate visuospatial task constraints. Exp Brain Res 211:231–242. 10.1007/s00221-011-2674-3 21503648

[B86] Yeo SH, Franklin DW, Wolpert DM (2016) When optimal feedback control is not enough: feedforward strategies are required for optimal control with active sensing. PLoS Comput Biol 12:e1005190. 10.1371/journal.pcbi.1005190 27973566 PMC5156370

